# Effects of the inspiratory muscle training and aerobic training on respiratory and functional parameters, inflammatory biomarkers, redox status and quality of life in hemodialysis patients: A randomized clinical trial

**DOI:** 10.1371/journal.pone.0200727

**Published:** 2018-07-26

**Authors:** Pedro Henrique Scheidt Figueiredo, Márcia Maria Oliveira Lima, Henrique Silveira Costa, Jeanne Brenda Martins, Olga Dumont Flecha, Patrícia Furtado Gonçalves, Frederico Lopes Alves, Vanessa Gomes Brandão Rodrigues, Emílio Henrique Barroso Maciel, Vanessa Amaral Mendonça, Ana Cristina Rodrigues Lacerda, Érica Leandro Marciano Vieira, Antônio Lúcio Teixeira, Fabrício de Paula, Cláudio Heitor Balthazar

**Affiliations:** 1 Programa Multicêntrico de Pós-Graduação em Ciências Fisiológicas-PMPGCF, Universidade Federal dos Vales do Jequitinhonha e Mucuri (UFVJM), Diamantina, Minas Gerais, Brazil; 2 Physical Therapy School, Universidade Federal dos Vales do Jequitinhonha e Mucuri (UFVJM), Diamantina, Minas Gerais, Brazil; 3 Medical School, Universidade Federal de Minas Gerais (UFMG), Belo Horizonte, Minas Gerais, Brazil; 4 Dentistry School, Universidade Federal dos Vales do Jequitinhonha e Mucuri, Diamantina, Minas Gerais, Brazil; 5 Hemodialysis Unit of the Santa Casa de Caridade de Diamantina Hospital, Diamantina, Minas Gerais, Brazil; 6 Medical School, Universidade Federal dos Vales do Jequitinhonha e Mucuri (UFVJM), Diamantina, Minas Gerais, Brazil; UFMG, BRAZIL

## Abstract

**Objective:**

Evaluate and compare the isolated and combined effects of Inspiratory Muscle Training (IMT) and Aerobic Training (AT) on respiratory and functional parameters, inflamatory biomarkers, redox status and health-related quality of life (HRQoL) in hemodialysis patients.

**Methods:**

A randomised controlled trial with factorial allocation and intention-to-treat analysis was performed in hemodialysis patients. Volunteers were randomly assigned to performe 8-weeks of IMT at 50% of maximal inspiratory pressure (MIP), low intensity AT or combined training (CT). Before the interventions, all the volunteers went 8-weeks through a control period (without training). Measures are taken at baseline, 8-week (after control period) and 16-week (after the interventions). Primary outcomes were functional capacity (incremental shuttle walk test), MIP and lower limbs strength (Sit-to-Stand test of 30 seconds). Plasma levels of interleukin-6 (IL-6), soluble tumor necrosis factor receptor 1 (sTNFR1) and 2 (sTNFR2), adiponectin, resistin and leptin, redox status parameters and HRQoL (KDQOL-SF questionnaire) were the scondary outcomes. Data analyses were performed by *two-*way *repeated measurements ANOVA*.

**Results:**

37 hemodialysis patients aged 48.2 years old (IC95% 43.2–54.7) were randomized. Increase of MIP, functional capacity, lower limbs strength and resistin levels, and reduction of sTNFR2 levels in 16-week, compared to baseline and 8-week, were observed in all the groups (p<0.001). IMT improved functional capacity, MIP and lower limbs strength in 96.7m (IC95% 5.6–189.9), 34.5cmH_2_O (IC95% 22.4–46.7) and 2.2repetitions (IC95% 1.1–3.2) respectively. Increase in resistin leves and reduction in sTNFR2 leves after IMT was 0.8ng/dL (IC95% 0.5–1.1) and 0.8ng/dL (IC95% 0.3–1.3), respectively, without between-group differences. Compared to baseline and 8-week, adiponectin levels (p<0.001) and fatigue domain of the HRQoL (p<0.05) increased in 16-week only in CT.

**Conclusion:**

IMT, AT and CT improved functional parameters and modulated inflammatory biomarkers, in addition, IMT provoked a similar response to low intensity AT in hemodialysis patients.

**Trial registration:**

Registro Brasileiro de Ensaios clínicos RBR-4hv9rs.

## Introduction

Abnormalities in muscle structure and function, such as a decrease in strength and endurance,[1] are common clinical findings in patients with End-stage Renal Disease (ESRD). These changes are determined by factors associated with the ESRD progression and hemodialysis treatment and a contribution to protein-energy wasting and uremic myopathy.[2] Among these factors, chronic inflammation and changes in the redox state are cited as determining factors for the alterations in energetic function and in the capacity of muscular contraction in ESRD, associated with the sedentary lifestyle.[2] Associations between inflammatory markers and redox state parameters with peripheral muscle weakness have been demonstrated in chronic kidney disease [3–6], as well as being predictors of death.[7, 8]

As happens in the muscles of the locomotor system, the function of the inspiratory muscles can also be impaired in patients with ESRD. In a recent study, the reduction in the inspiratory muscle strength, evaluated by maximum inspiratory pressure (MIP), was demonstrated in a sample from hemodialysis patients. In addition, the inspiratory muscle weakness was an independent predictor of functional capacity impairment in this population.[9] Thus, inspiratory muscle training (IMT) could improve functional capacity in hemodialysis patients.[10]

The efficacy of IMT in the functional capacity improvement has already been described in other health conditions.[11] In ESRD, evidence for this therapeutic modality in increasing MIP and functional capacity is based on the results of a few studies.[12–15] Furthermore, the magnitude of IMT, compared to a conventional aerobic training (AT) modality, are not known. Thus, the objectives of the present study were to evaluate and compare the isolated and combined effects of the IMT and low intensity AT on nutritional, respiratory and functional parameters, inflammatory biomarkers and redox status, as well as on the health-related quality of life (HRQoL) domains of hemodialysis patients. The alternative hypothesis this sudy was that the improvements induced by IMT are lower than that induced by low intensity AT and that the combined IMT and AT induce greater improvements than the isolated approaches.

## Materials and methods

### Study design

In this randomised controlled trial with factorial allocation and intention-to-treat analysis, participants were randomly allocated into three experimental condictions: IMT, AT or combined training (CT). After selection of participants, the volunteers were stratified by hemodialysis schedule (7:00h, 12:30h e 16:30h). Randomisation was performed using individual allocation codes placed within opaque, sealed envelopes by a person having no contact with the participants. Potential participants were screened to verify eligibility before baseline assessment and randomisation. The research was carried out in accordance with the declaration of Helsinki and was approved by the ethics committee of the Centro Universitário UNA-BH (protocol 37412314.5.0000.5098). All the patients gave their written, informed consent before participating in the study.

### Participants

Between January 2015 and December 2015, ESRD patients receiving hemodialysis treatment in the Santa Casa de Caridade de Diamantina Hospital were selected. To be included in the study, patients had to be more than 18 years old. They should not be receiving anti-inflammatory or antiallergic medication, under hemodialysis treatment three times a week for at least three months, and with arteriovenous fistula for hemodialysis access. Exclusion criteria were any contraindication to physical exercise or inability to perform the functional tests. The sample size was calculated a priori, according to the procedure proposed by Jakel et al (2005) for repeated measures [16]. Considering functional capacity variations of 47% between IMT and lower limb training, standard deviation of 30%,[13] power of 80% and alpha of 5% (two-tailed). The sample size was estimated in 10 volunteers per group.

### Interventions

All interventions (IMT, AT and CT) were intradialitic, and they were performed during the first two hours of dialysis, three times a week for eight weeks or 24 sessions. The sessions lost by the volunteers were not restored. Thus, adherence was recorded by the number of sessions held in the 24 offered.

IMT was performed using Threshold IMT® (Respironics, Murrysville PA, USA) or PowerBreathe light or median Resistance (Powerbreathe, HaB International Ltd, Southam, UK), according to the previously evaluated MIP. Participants performed three sets of 15 deep inspirations at the equipment mouthpiece and rested for 60 seconds with the linear load adjusted to 50% of MIP.[13] MIP was reevaluated every six sessions for load adjustment.

The AT was performed by cycle ergometer (Mini Bike E5, ACTE Sports, São Paulo, Brasil) positioned in the front of patients’ chairs. The cycling session consisted of a 5-minute warm-up, 30 minutes of cycling at target workload, and a 5-minute cooling-down period. During exercise, patients were asked every 5 minutes about the fatigue score, and the cycle ergometer load was adjusted to achieve a fatigue score between three and five points in the modified Borg Scale. The speed remained ≥ 50 rpm.[17]

In the CT sessions, IMT was performed immediately before AT and, in the AT group, the participants performed sets of inspirations with IMT devices, but without resistance to inspiration (Sham-IMT). The eight weeks prior to initiating the interventions were considered to represent the control period. During eight weeks prior to initiating the interventions the volunteers did not receive any type of training to represent the control period. The dialysis prescription and medication therapy remained unchanged during the study.

After medical and dental evaluation, the volunteers were evaluated in the baseline, 8-week follow-up (after control period) and 16-week follow-up (after intervention period), on dialitic days, immediately before the hemodialysis sessions, except for the evaluation of body composition.

### Outcome measures

#### Primary outcomes

**Maximum inspiratory pressure.** With the volunteers seated, MIP were evaluated with a calibrated aneroid manovacuometer (MV-150/300, Ger-Ar, São Paulo, Brazil), equipped with a 2-mm-diameter hole in the nozzle, from residual volume. The highest value of three valid measurements was considered.[18] Maximum predicted values were estimated.[19]

**Functional capacity.** The functional capacity was evaluated by the Incremental Shuttle Walk Test (ISWT).[20, 21] Volunteers were instructed to walk or run in a 10 m corridor and the minimum speed was determined by an audio signal. The ISWT has 12 progressive intensity levels, and the test was terminated when the volunteer failed to reach the minimum speed required on a level surface two consecutive times.[22, 23] The distance walked was recorded, and the predicted values were estimated.[24]

**Lower limbs strength.** The lower limbs strength was assessed by the Sit-to-Stand test of 30 seconds (SST).[25] Volunteers were instructed to stand up completely from a 45 cm chair and sit down as quickly as possible until the volunteer touched the back on the backrest, with no support from the upper limbs. The best performance (number of full movements in 30 seconds) of two tests with a minimum interval of 5 minutes between them was considered. No verbal encouragement was provided.

#### Secondary outcomes

**Inflammatory biomarkers.** Ethylenediaminetetraacetic acid vacutainers (BD vacutainers, Franklin Lakes, NJ, USA) were used to collect 8-ml venous blood samples immediately before the second weekly hemodialysis session (midweek), without fasting. The tubes was centrifuged at 2500 rpm for 10 min at 4°C and were stored as plasma and erythrocyte aliquots at -80°C until use. Plasma soluble tumor necrosis factor receptor 1 and 2 (sTNFR1 and sTNFR2, respectively), leptin, adiponectin and resistin levels were measured using conventional sandwich ELISA kits (DuoSet, R&D Systems, Minneapolis, MN, USA) according to the manufacturer’s instructions. The detection limits were 5.0 pg/mL for all the kits. The plasma interleukin 6 (IL-6) level was measured using the cytometric bead arrays kit (BD Bioscience, San Jose, CA, USA) according to the manufacturer’s protocol. Samples were acquired in a FACSCanto flow cytometer (BD Biosciences, San Jose, CA, USA) and analyzed using the FCAP Array v1.0.1 software (Soft Flow Inc.).

Measurements of redox status. The redox status was evaluated by the plasmatic level of the thiobarbituric-acid-reactive substances (TBARS) and ferric reducing antioxidant power (FRAP), as well as by use of the antioxidant enzymes dismutase superoxide (SOD) and catalase (CAT) activity in the erythrocyte lysate. The erythrocyte lysate was prepared as described by Glass and Gershon.[26] The protein concentration of samples was determined by the Bradford method[27] using bovine serum albumin (BSA) (1 mg/mL) as the standard. TBARS levels were measured according to the method described by Ohkawa et al.,[28] and the reactions between thiobarbituric acid and malondialdehyde (MDA) was used to determine the degree of lipid peroxidation. The total antioxidant capacity (FRAP) was determined according to the method of Benzie and Strain.[29] The assay to determine SOD activity was performed according to Srivastava et al.[30] and expressed in U/mg of protein. The CAT activity was measured according to the method of Nelson and Kiesov[31] and expressed as ΔE/min/mg protein, where ΔE represents the variation in enzyme activity during one minute.

**Health-related quality of life (HRQoL).** The HRQoL was evaluated by a specific questionnaire—Kidney Disease Quality of Life (KDQOL-SF).[32]

**Anthropometric/physical Parameters.** The Anthropometric/physical parameters were blind and evaluated imediatelly after the second weekly hemodialiysis session. Weight, body mass index (BMI), waist circunference and body fat percentage were evaluated. Body fat percentage was evaluated by an analogic plicometer (Sanny, American Medical do Brasil, Brasil). Three sinkfolds (tricipital, suprailiac and abdominal in womans and tricipital, subscapular and middle pectoral in mens) were measured on the right side. The percentage of fat mass was obtained by use of the equations of Jackson & Pollock (1985)[33] and Siri (1961).[34] The measurement of inflammatory biomarkers, redox status and Anthropometric/physical parameters were blinded, performed by a person having no contact with the participants.

### Statistical analysis

The Sigma Stat (Version 9.0) statistic program was used for the statistical analysis. The normality of data were verified using the Shapiro-Wilk test. Categorical variables are presented as absolute and relative frequencies, and continuous variables are presented as the mean (IC95%). Categorical variables were compared by the Chi-squared test. The effects of the interventions were compered by Two-Way Repeated Measures ANOVA (within-group, between-group and interactions) with Tukey’s post hoc test. For variables with a statistical significance to the within-group differences, the comparisons of the 8-week to 16-week variations (intervention period) was performed by the one-way ANOVA or Kruskall Wallis test, with Tukey’s post hoc test. The significance level was set at 0.05. For reasons of cost, secondary outcomes were assessed only in participants who completed all steps of the treatment originally allocated (Per-protocol analysis).

## Results

### Flow of participants through the study

**Fig 1:**
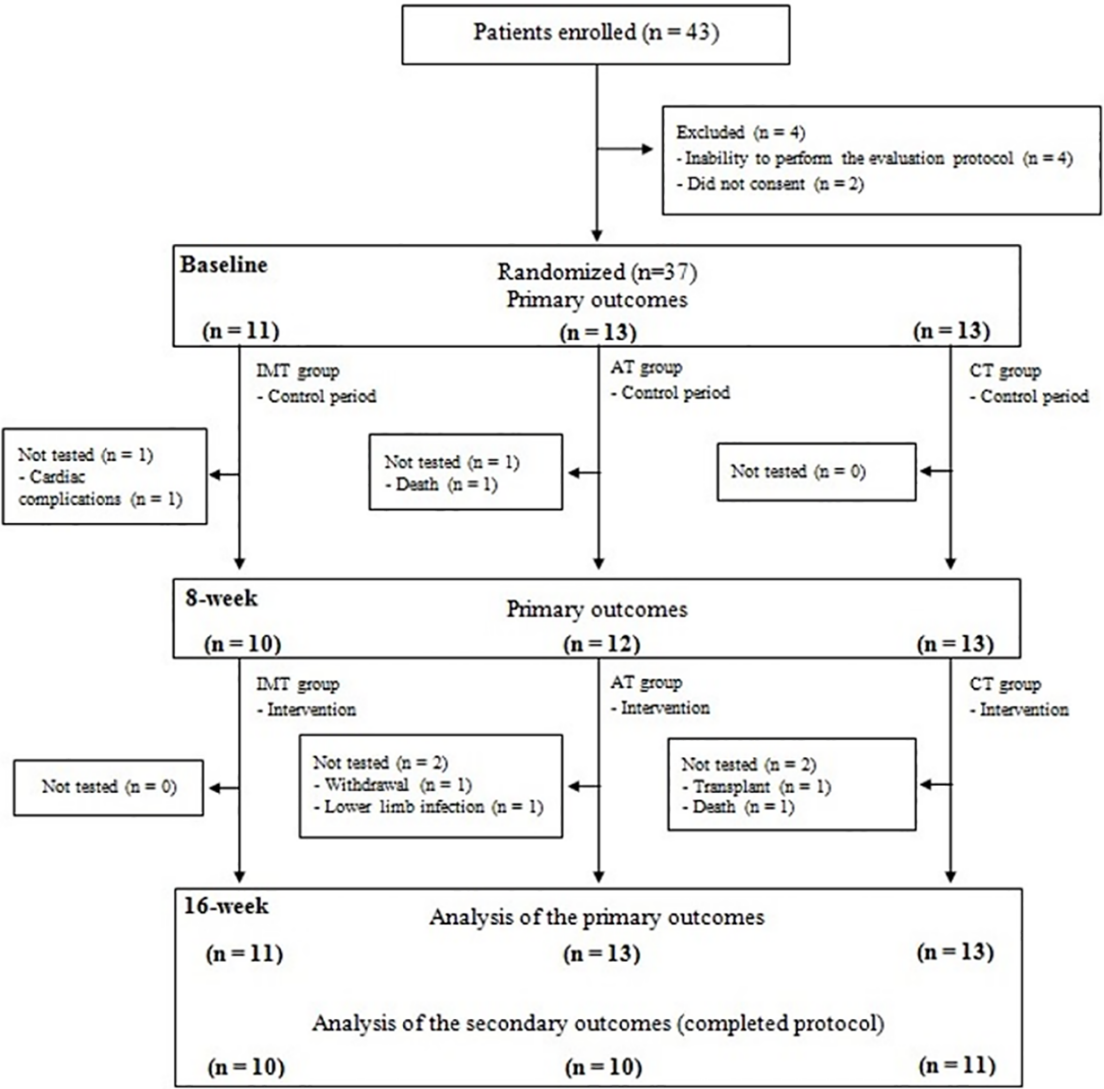
Flow chart. IMT: inspiratory muscle training; AT: aerobic training; CT: combined training.

Out of a total of 93 ESRD patients, 43 were selected and 37 were randomized (11 in IMT group, 13 in AT group and 13 in CT group). The flow of the participants through the study is illustrated in Fig 1. The participants’ demographics and clinical characteristics are presented in Table 1. The three groups were similar at the baseline. The volunteers were predominantly male (70.3%) and had a mean age of 48.2 years (IC95% 44.5–51.9) and dialysis data, kt/v indexes of 1.5 (IC95% 1.4–1.7) and urea reduction rate of 70.8% (IC95% 67.0–74.7), demonstrated the efficiency of hemodialysis treatment.

**Table 1:** Characteristics of the sample according to treatment allocation.

	IMT (N = 11)	AT (N = 13)	CT (N = 13)	p
**Sex, n%**				
**Male**	7 (63.6)	10 (76.9)	9 (69.2)	0.77
**Female**	4 (36.4)	3 (23.1)	4 (30.8)	
**Race, n%**				
**Caucasian**	5 (45.5)	6 (46.1)	5 (38.4)	0.64
**Black**	5 (45.5)	4 (30.8)	4 (30.8)	
**Other**	1 (9.0)	3 (23.1)	4 (30.8)	
**Age, yars**	52.8 (43.1–62.5)	49.5 (41.6–57.3)	45.2 (34.8–55.5)	0.46
**Weight, kg**	67.7 (54.9–80.5)	69.1 (57.5–80.8)	64.5 (52.9–76.2)	0.82
**BMI, kg/m**^**2**^	25.1 (21.1–29.2)	25.2 (21.5–28.8)	24.2 (20.9–27.2)	0.85
**Waist, cm**	86.2 (73.4–98.9)	87.9 (77.4–98.4)	84.5 (73.9–95.2)	0.89
**ESRD etiology, n%**				
**Hypertensive nephropathy**	4 (36.4)	3 (23.1)	6 (46.1)	
**Diabetic nephropathy**	2 (18.2)	3 (23.1)	3 (23.1)	
**Glomerulonephritis**	2 (18.2)	1 (7.7)	1(7.7)	
**Polycystic kidneys**	0	1 (7.7)	0	
**Others**	3 (27.2)	5 (38.4)	3 (23.1)	
**Comorbity condictions, n%**				
**Tabagism**	2 (18.2)	1 (7.7)	3 (23.1)	0.56
**Ethylism**	0	0	0	**——**
**Diabetic**	2 (18.2)	2 (15.4)	3 (23.1)	0.88
**Obesidity**	2 (18.2)	2 (15.4)	3 (23.1)	0.88
**Periodontal disease, n%**				
**Periodontal health**	7 (63.6)	7 (53.9)	8 (61.5)	0.80
**Gengivitis**	2 (18.2)	5 (38.4)	3 (23.1)	
**Mild disease**	2 (18.2)	1(7.7)	2 (15.4)	
**Duration of dialysis, years**	4.4 (1.4–7.5)	3.0 (0.5–6.4)	4.9 (2.7–7.1)	0.30
**kt/v**	1.7 (1.4–1.7)	1.6 (1.4–1.7)	1.5 (1.3–1.7)	0.74
**Urea reduction ratio, %**	70.5 (66.6–74.4)	72.4 (68.6–76.2)	71.7 (67.2–76.1)	0.70
**Hb, mg/dL**	10.9 (9.8–11.8)	10.3 (9.4–11.2)	10.9 (10.3–11.5)	0.52
**Hematócrit, %**	34.4 (31.2–37.5)	32.1 (29.6–34.6)	33.8 (31.8–35.9)	0.37
**Albumin, g/dL**	3.7 (3.5–3.9)	3.8 (3.6–4.0)	3.9 (3.7–4.0)	0.43
**Beginning of dialysis, n(%)**				
**7:00h**	3(27.2)	2 (15.4)	4 (30.8)	0.81
**12:30h**	3(27.2)	6 (46.2)	5 (38.4)	
**16:30h**	5(45.6)	5 (38.4)	4 (30.8)	

Data represented as mean (IC95%) or n (%). IMT: inspiratory muscle training group; AT: aerobic training group; CT: combined training group; BMI: body mass index; ESRD: End-Stage Kidney Disease; Kt/V:dialysis efficiency; Hb: hemoglobin.

### Adherence to the study protocol

The number of the execise sessions in the IMT, AT and CT groups were 23.1 (22.5–24.0), 23.1 (IC95% 22.5–24.0) and 22.7 (IC95% 21.8–23.6), respectively (p = 0.279). This number representes an assiduity of 96.4% (IC95% 93.3–99.6), 96.4% (IC95% 93.0–99.8) and 94.9% (IC95% 91.3–98.5), respectively (p = 0.279). Two participants, one of the IMT group and one of the AT group, did not participate in the 8-week and 16-week follow-up assessments. The data of these patients were imputed on analysis, repeating the results of baseline assesment in the 8-week and 16-week follow-up. During intervention periods, two participants of the AT group and two volunteers of the CT group were not evaluated in the 16-week follow-up assessment. Data were imputed for these participants using the mean of the baseline and 8-week measurements in 16-week follow-up. The results of the study were similar when these values were not imputed.

The interventions performed in the AT group and the CT group were low intensity physical training programs with fatigue scores at 3.3 (IC95% 2.4–4.1) and 3.0 (IC95% 2.4–3.5), respectivey, without any differences between them (p = 0.701). However, their fatigue scores were higher than those observed in the IMT group (0.3 (IC95% 0.0–0.5); p < 0.001). The whole duration of the IMT sessions did not exceed 10 minutes and no adverse effects associated to the interventions were observed.

### Primary outcomes

**Fig 2:**
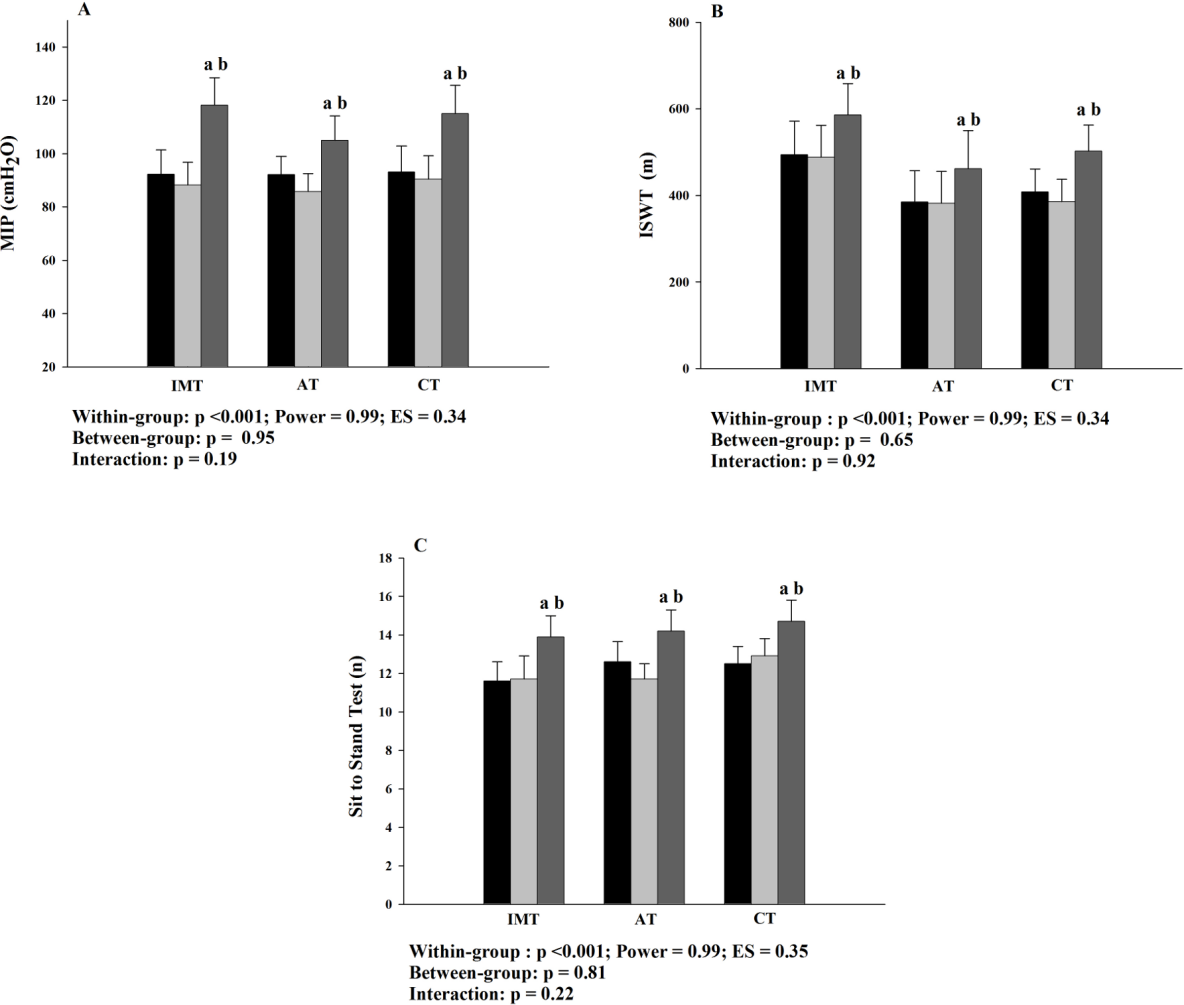
Effects of the interventions in primary outcomes. A: inspiratory muscle strength; B: functional capacity; C: lower limb strength; MIP: máximal inspiratory pressure; ISWT: incremental shuttle walk test: IMT: inspiratory muscle training; AT: aerobic training; CT: combined training; ES: effect size; ■ Baseline; ■ 8-week; ■ 16-week. Baseline to 8-week: control period; 8-week to 16-week: intervention period. a: p<0.05 to baseline; b: p<0.05 to 8-week.

The effects of the interventions in primary outcomes are demonstrated in Fig 2. Was shown within-group difference, with an increase of the inspiratory muscle strength (Fig 2A), functional capacity (Fig 2B) and lower limb strength (Fig 2C) in 16-week, compared to baseline and 8-week. There were no between-group differences. The MIP increased 34.5 cmH_2_O (IC95% 22.4–46.7) after IMT (8-week to 16-week). This increase was similar (p = 0.06) to AT and CT group (18.5 cmH_2_O (IC95% 9.9–27.0) and 26.9 cmH_2_O (IC95% 17.2–36.7), respectively). The increases in functional capacity after IMT, AT and CT were 96.7 m (IC95% 5.6–187.9), 86.6 m (IC95% 21.8–151.5) and 147.7 m (IC95% 94.1–201.3), respectively (p = 0.251), as well the lower lib strength incresed in 2.2 repetition (IC95% 1.1–3.2), 3.1 repetition (IC95% 2.1–4.1) and 2.4 repetition (IC95%1.4–3.5) after IMT, AT and CT, respectively (p = 0.671). No interaction was found and there were no statistical differences during the control period for the variables evaluated.

### Secondary outcomes

The results of the interventions on anthropometric/physical parameters are presented in Table 2. There were no significant differences for the anthropometric/physical variables evaluated. Similar results were found for redox status parameters (Table 3).

**Table 2:** Effects of the interventions on anthropometric/physical parameter.

Varibles	IMT (n = 10)	AT (n = 10)	CT (n = 11)	Within- group	Between-group	Interaction
				p	p	p
**Weight, kg**				0.201	0.713	0.451
Baseline	70.0 (53.6–82.3)	71.9 (58.6–85.2)	64.6 (50.6–78.6)			
8-week	68.7 (54.3–83.0)	71.7 (59.0–84.3)	65.2 (51.6–78.8)			
16-week	68.5 (54.1–82.9)	72.2 (59.6–84.8)	64.9 (51.5–78.3)			
**BMI, kg/m**^**2**^				0.628	0.905	0.451
Baseline	25.2 (20.7–29.6)	25.8 (21.2–30.5)	24.2 (20.6–27.8)			
8-week	25.4 (21.0–29.8)	26.2 (22.2–30.3)	24.4 (20.9–27.9)			
16-week	25.3 (20.9–29.8)	26.4 (22.3–30.4)	24.3 (20.9–27.8)			
**Waist, cm**				0.090	0.744	0.181
Baseline	85.1 (71.0–99.2)	89.5 (77.2–101.7)	83.4 (71.8–94.9)			
8-week	85.9 (71.3–100.4)	90.3 (77.8–102.8)	83.6 (72.7–94.4)			
16-week	86.1 (71.9–100.3)	87.4 (74.6–100.2)	82.3 (72.1–92.4)			
**Body fat, %**				0.168	0.501	0.944
Baseline	16.7 (12.8–20.5)	18.6 (13.6–23.3)	15.4 (8.5–22.3)			
8-week	16.8 (12.9–20.6)	18.7 (8.7–22.3)	15.5 (8.7–22.3)			
16-week	16.7 (12.8–20.6)	18.8 (13.9–23.6)	15.5 (8.7–22.4)			

Data represented as mean (IC95%). IMT: inspiratory muscle training group; AT: aerobic training group; CT: combined training group; BMI: body mass index; Baseline to 8-week: control period; 8-week to 16-week: intervention period.

**Table 3:** Effects of the interventions on redox status parameters.

Varibles	IMT (n = 10)	AT (n = 10)	CT (n = 11)	Within-group	Between-group	Interaction
				p	p	p
**SOD, U/mg protein**				0.58	0.63	0.75
Baseline	27.3 (13.1–41.6)	16.0 (12.1–19.9)	24.1 (8.1–40.1)			
8-week	22.2 (12.7–31.8)	14.3 (8.06–20.6)	25.1 (15.4–34.9)			
16-week	22.3 (13.8–30.7)	17.3 (13.4–21.2)	24.4 (14.5–34.3)			
**Catalase, ΔE/min/mg**				0.62	0.37	0.88
Baseline	36.7 (31.2–42.2)	26.5 (19.2–33.8)	34.9 (24.1–48.7)			
8-week	39.7 (29.2–50.2)	29.5 (18.7–40.3)	40.5 (28.9–52.1)			
16-week	42.2 (31.4–52.9)	25.8 (18.4–33.1)	38.2 (29.1–47.2)			
**TBARS, nmol MDA/μg protein**				0.37	0.45	0.37
Baseline	0.3 (0.1–0.4)	0.2 (0.1–0.2)	0.2 (0.1–0.3)			
8-week	0.2 (0.1–0.4)	0.2 (0.1–0.2)	0.2 (0.1–0.3)			
16-week	0.1 (0.0–0.2)	0.2 (0.1–0.2)	0.2 (0.1–0.4)			
**FRAP, mmolFeSO**_**4**_**/L/mg protein**				0.57	0.18	0.32
Baseline	49.2 (29.7–68.6)	41.5 (34.6–48.3)	36.8 (31.0–42.7)			
8-week	46.8 (29.9–63.6)	44.7 (35.1–54.4)	38.2 (34.3–42.0)			
16-week	35.9 (27.2–44.6)	43.1 (36.0–50.1)	36.6 (24.4–46.8)			

Data represented as mean (IC95%). IMT: inspiratory muscle training group; AT: aerobic training group; CT: combined training group; SOD: superoxide dismutase; TBARS: thiobarbituric-acid-reactive substances; FRAP: ferric reducing antioxidant power: Baseline to 8-week: control period; 8-week to 16-week: intervention period.

**Fig 3:**
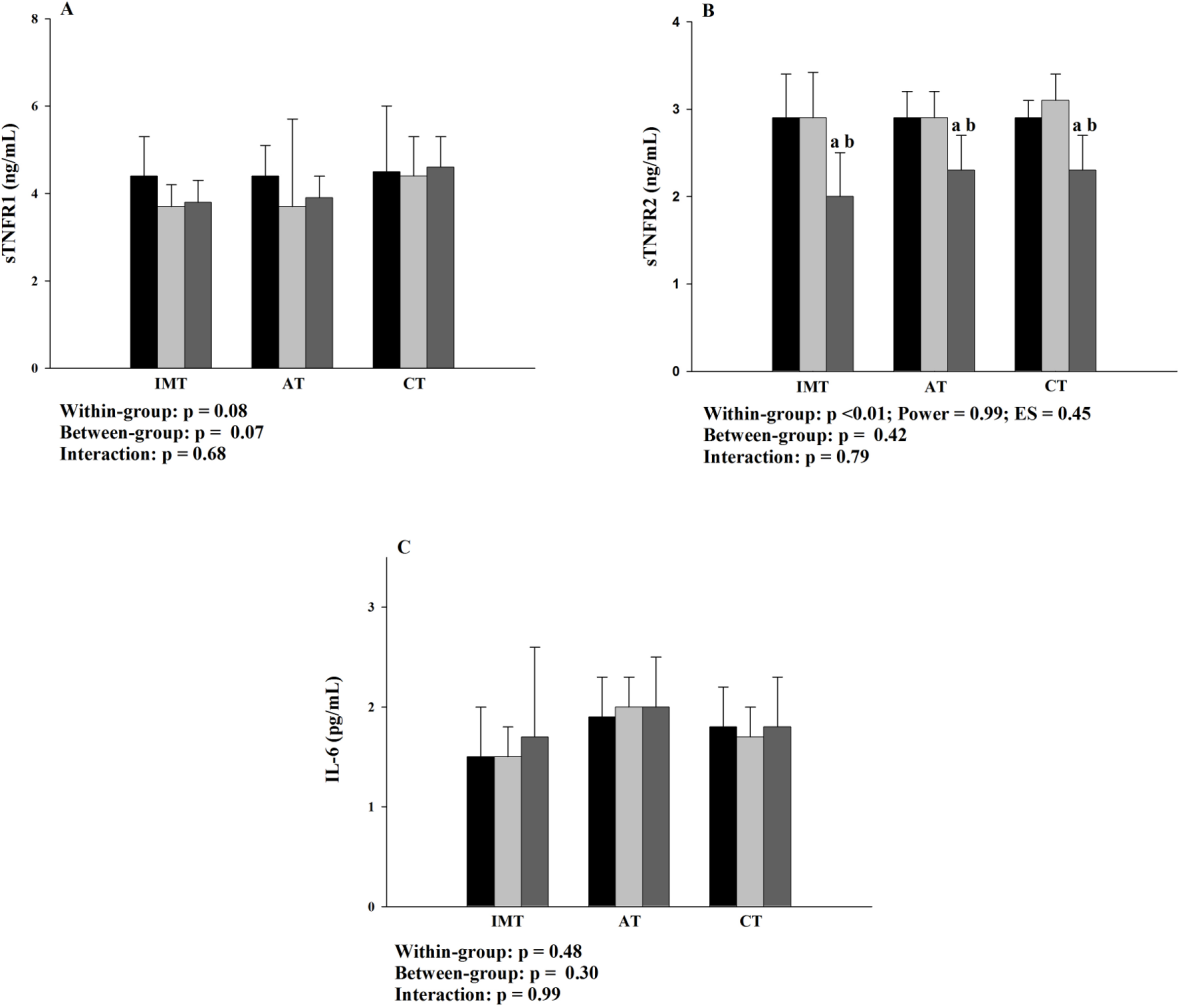
Effects of the interventions on plasma sTNFR1, sTNFR2 and IL-6 levels. A: plasma sTNFR1 levels; B: plasma sTNFR2 levels; C: plasma IL-6 levels; sTNFR1 and 2: soluble receptors 1 and 2, respectively, of the tumor necrosis alpha factor; IL-6: interleukin 6; IMT: inspiratory muscle training group; AT: aerobic training group; CT: combined training group; ES: effect size; ■ Baseline; ■ 8-week; ■ 16-week. Baseline to 8-week: control period; 8-week to 16-week: intervention period. a: p<0.05 to baseline; b: p<0.05 to 8-week.

**Fig 4:**
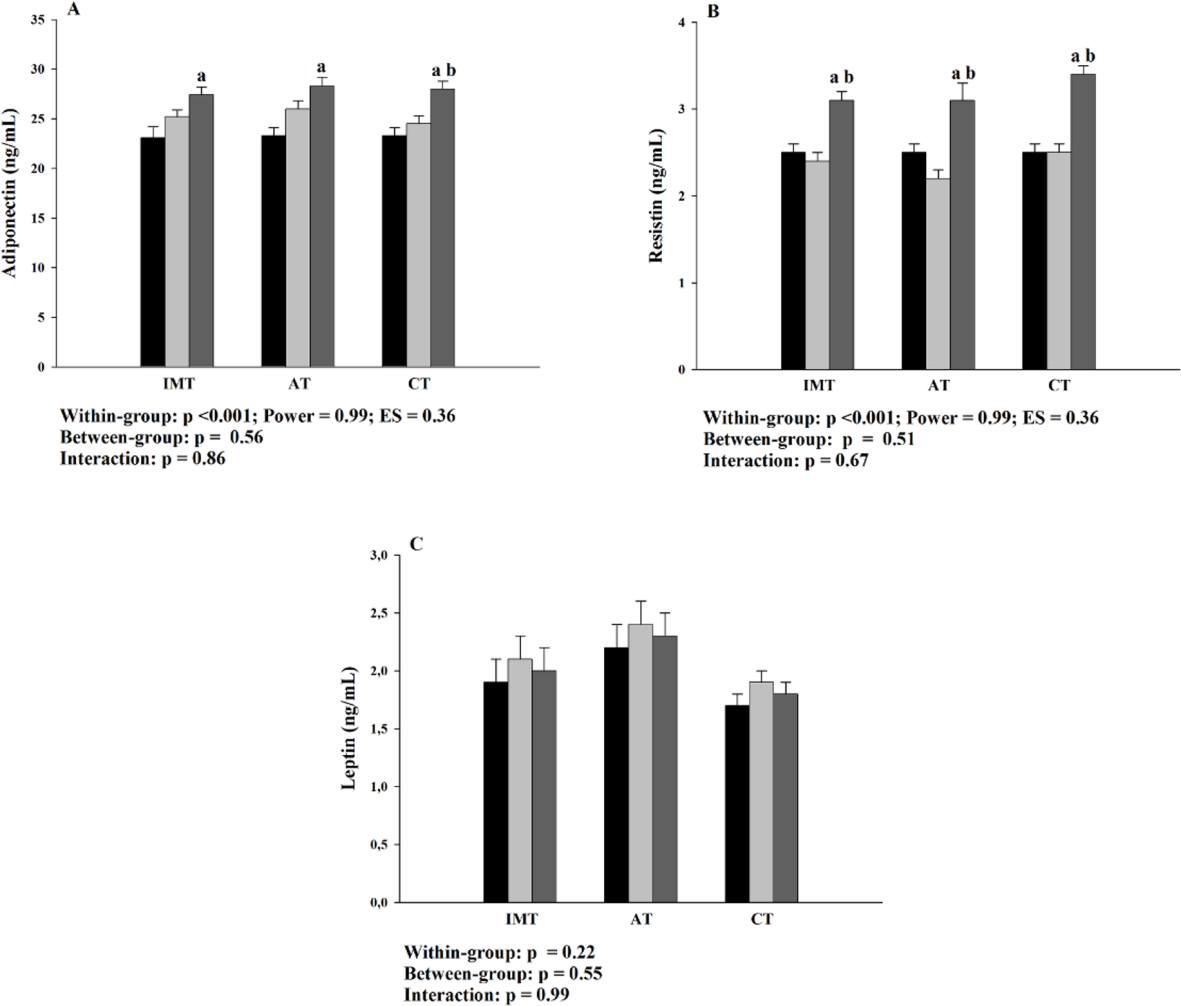
Effects of the interventions on plasma adipokines levels. A: plasma adiponectin levels; B: plasma resistin levels; C: plasma leptin levels; IMT: inspiratory muscle training group; AT: aerobic training group; CT: combined training group; ES: effect size; ■ Baseline; ■ 8-week; ■ 16-week. Baseline to 8-week: control period; 8-week to 16-week: intervention period. a: p<0.05 to baseline; b: p<0.05 to 8-week.

The effects of the interventions on inflammatory biomarkers are illustrated in Fig 3 and Fig 4. Reductions in plasma sTNFR2 levels (Fig 3B) were found after interventions in all the groups, when compared to baseline and 8-week (p<0.001). The reductions in plasma sTNFR2 were 0.8 ng/dL (IC95% 0.3–1.3), 0.5 ng/dL (IC95% 0.2–0.8) and 0.7 ng/dL (IC95% 0.5–1.1) after IMT, AT and CT, respectively (p = 0.393). There were no between-group differences or interactions for plasma sTNFR2 and there were no significant differences for the sTNFR1and Il-6 (Fig 3A and Fig 3C, respectively).

There were increase in all groups in adiponectin levels in 16-week, compared to baseline (Fig 4A). However, the intervention-associated effect (8-week to 16-week) was only found after CT. The increase of the adiponectin levels after CT was 3.4 ng/dL (IC95% 1.1–5.7). Simillary, within-group differences was shown for plasma resistin levels (Fig 4B). There were increases of the resistin levels in 16-week, relative to baseline and 8-week in all the interventions. Plasma resistin levels increased in 0.7 ng/dL (IC95% 0.5–1.1), 0.9 ng/dL (IC95% 0.5–1.3) and 0.8 ng/dL (IC95% 0.2–1.4) after IMT, AT and CT, respectively (p = 0.875). There were no between-group differences or interactions for plasma adiponectin or resistin levels and no significant differences were found for leptin levels (Fig 4C).

The results of the interventions in HRQoL domains are showed in S1 Table. Was observed within-group differences in the “energy/fatigue” domain, with an increase in the scores 16-week relative to 8-week and baseline. Through post hoc analysis, these effects were only found after CT. Similar results was shown for scores of the “physical funtioning” and “role physical” domains. However, the individual effects of interventions were not observed through post hoc analysis, as well as for “emotional being health” domains. Finally, the scores of the “pain” domain increased during the control period before IMT. There was no statistical significance for other domains of the KDQOL-SF questionnaire.

## Discussion

To the best of our knowledge, this is the first study that compared the effects of IMT, performed in isolation and combined with aerobic training, on anthropometric/physical, respiratory and functional parameters, inflammatory biomarkers, redox status and HRQoL in people with ESRD undergoing hemodialysis. The main findings of the present study were: (1) the effects of IMT on the evaluated parameters were similar to those of low intensity AT; and (2) the addition of IMT to AT increases adiponectin levels and improves physical domains of HRQoL. The present study suggests that the IMT, a low-cost, easy-to-perform training that does not require physical space, can be a therapeutic model in the hemodialyis units when conventional AT can not be implemented.

The effects of IMT on the MIP were similar to those of previous studies.[12, 13, 15] However, Silva et al.[14] did not find significant changes in MIP after IMT. The intensity of IMT performed by the authors was lower than that of the present study. In the comparison among groups, no differences were demonstrated, suggesting a similar increase in MIP after IMT, AT and CT. In addition, the increase in MIP after all interventions was greater than the minimal clinically important difference in individuals with chronic respiratory disease.[35] Considering that the MIP is an independent preditor to functional capacity in hemodialysis patients, [9] increases of the distance covered during ISWT would be expected. In fact, we also demonstrated the increase in functional capacity after all three interventions. The increase in functional capacity after IMT was higher than the minimal clinically important difference proposed for the ISWT (70 m) in cardiovascular rehabilitation [36] and for pearsons with chronic kidney disease (60 m). [37] The improvement in functional capacity after interventions was similar between-group. This finding has an important clinical meaning, because IMT increased functional capacity in magnitude similar to conventional AT. A similar clinical meaning can be attributed to the strength of the lower limb muscles because the sit-to-stand test is cited as a test for the functional evaluation of people with ESRD.[1] Although the specific values of minimal detectable change for this test for hemodialysis patients are not know, the increase of the SST after IMT was higher than they showed by others studies, after differents physical training programs.[38, 39]

To date, the effects of IMT on inflammatory biomarkers in hemodialysis patients have been evaluated in only one study.[13] The authors demonstrated reductions in C-reactive protein after intervention. Regarding sTNFR, this is the first study to evaluate the effects of IMT in this population, and the effects of physical training programs have been little explored. In 2014, Viana et al.[40] demonstrated that the sTNFR1 level decreased after six months of home-based AT, without sTNFR2 changes, a result contrary to that observed in our study. This result may be due to the difference in the volume of training. In addition, the sample studied by the authors was composed of older individuals. It is important to consider that the reduction of the sTNFR2 level might be clinically relevant because it is determinant for manifestation of erythropoietin resistance in ESRD,[41] as well as being an independent predictor for death.[7] In the analysis of the other inflammatory biomarkers, the levels of sTNFR1 and IL-6 were lower than those observed by other authors,[7, 40, 41] probably because of the recruitment of younger, a shorter time of dialysis treatment than in the other studies or both.

In addition to the reduction in sTNFR2, there was an increase in adiponectin level, especially after CT. This result suggests a positive effect of the combination of IMT and AT on the modulation of anti-inflammatory activity, considering the role of adiponectin in the immune system and in cardioprotection.[42] Also, all three interventions increased resistin levels. The effects of exercise training on resistin levels are poorly understood. Although it is traditionally associated with increased insulin resistance and pro-inflammatory activity,[43] increased levels after physical training in healthy men[44] and higher levels in athletes[45] have been demonstrated. This response could be related to the increase in the lipid metabolism in the exercise muscle,[45] to an antioxidant action of resistin induced by the training[43] or to a potential cross-talk between resistin and signaling of an insulin/insulin-like growth factor.[46] In addition, low resistin levels have been associated with poor hospitalization-free survival in hemodialysis patients.[47] Although the mechanisms involved in these associations remain unknown, the effects of interventions on the levels of resistin, sTNFR2, and adiponectin suggest a positive modulating effect on the attenuation of the inflammatory process.

Regarding the leptin level, no significant changes after intervention were observed, possibly because there were no changes in the body composition of the volunteers. The low training volumes might also justify the results of the redox status parameters, considering that the improvement of the antioxidant capacity induced by the exercise training is directly dependent on the training volume and the target population.[48] For example, in the study by Wiuld and colleagues,[49] the moderate aerobic training performed during four months reduced TBARS from eight subjects, mostly obese, in hemodialysis treatment.

The global effects of interventions on inflammatory biomarkers can be considered clinically relevant. This assumption is based on the observed results in the sTNFR2, adiponectin and resistin levels. In the present study, the reduction of sTNFR2 levels, an independent predictor of death in ESRD,[7] occurred concomitantly with increasing of adiponectin and resistin levels. In ESRD, the increased of the adiponectin (especially after CT) and resistin levels, without changes in body composition and redox status, may result in a positive modulatory effect.[50, 51] Although the long-term clinical and functional effects of the observed results are unknown, the clinical influence of the biomarkers evaluated in the ESRD [7, 41, 42, 47] make the results of this work relevant. However, the effects of a long duration physical training programs on these variables need to be studied in this population.

In the setting of the HRQoL, the effects of IMT were previously reported[13], and a greater variation of the domains related to physical aspects after 10 weeks of IMT was demonstrated. The effects of AT are well established.[52] In the present study, at baseline, higher values were observed in most domains of the KDQOL-SF, which can be partially explained by the characteristics of the sample, considering the association between age and HRQoL.[53] Together with the low training volume, the characteristics of the sample might have contributed to the absence of significant effects. However, the score of the “energy/fatigue” domain increased in CT, which demonstrated an additional effect of the inclusion of IMT in AT. Thus, HRQoL results suggest that the applied interventions positively influence factors related to physical aspects, as reported in a recent meta-analysis.[52]

The present study demonstrated that the IMT can promote positive effects on respiratory, functional, inflammatory and HRQoL aspects in magnitudes similar to that of low intensity AT. Thus, the IMT, a low-cost and easy-to-perform training, can be a therapeutic model in the hemodialyis units when conventional AT can not be implemented. Interestingly the applied protocols presented a high adherence, which can be explained by the low intensity of the exercises. This highlights the applicability of the applied training modalities.

However, some issues need to be elucidated for the routine IMT application in dialysis centers: Which subgroup of patients would most benefit from IMT? What is the clinical meaning of IMT performed in a long-term setting? The elucidation of these questions can help in the elaboration of specific guidelines for IMT performance in hemodialysis patients.

As limitations, the lung volumes and capacities were not evaluated, and it was not possible to determine the degree of respiratory impairment in our sample. Also, our sample was younger and had fewer comorbidities than other studies.

We can conclude that eight weeks of IMT improved functional and inflammatory parameters of patients in hemodialysis treatment similarly to the effect of a low intensity AT. Although the combination of the two therapeutic modalities did not increase the functional capacity more expressively, improvements in the self-perception of HRQoL, especially in physical aspects, would justify the combined use of these therapies in ESRD patients undergoing hemodialysis.

## Supporting information

S1 Table Effects of the interventions on health related quality of life. (DOCX)

S1 File CONSORT checklist. (PDF)

S2 File Study protocol. (PDF)

S3 File Committee approval documents. (PDF)

## References

[1] Painter P, Marcus RL. Assessing physical function and physical activity in patients with CKD. Clin J Am Soc Nephrol. 2013; 8(5):861–72. 10.2215/CJN.06590712 23220421

[2] Chen CT, Lin S-H, Chen J-S, Hsu Y-J. Muscle wasting in hemodialysis patients: new therapeutic strategies for resolving an old problem. ScientificWorldJournal. 2013;5:2–7. 10.1155/2013/643954 PMC3870868 24382946

[3] Johansen KL, Shubert T, Doyle J, Soher B, Sakkas GK, Kent-Braun JA. Muscle atrophy in patients receiving hemodialysis: effects on muscle strength, muscle quality, and physical function. Kidney Int. 2003;63:291–297. 10.1046/j.1523-1755.2003.00704.x 12472795

[4] Wang AY, Sea MM, Tang N, Sanderson JE, Lui S-F, Li PK, et al Resting energy expenditure and subsequent mortality risk in peritoneal dialysis patients. J Am Soc Nephrol. 2004;15:3134–3143. 10.1097/01.ASN.0000144206.29951.B2 15579517

[5] den Hoedt CH, Bots ML, Grooteman MP, van der Weerd NC, Penne EL, Mazairac AH, et al Clinical predictors of decline in nutritional parameters over time in ESRD. Clin J Am Soc Nephrol. 2014;9:318–325. 10.2215/CJN.04470413 24458074 PMC3913235

[6] Beetham KS, Howden EJ, Small DM, Briskey DR, Rossi M, Isbel N, et al Oxidative stress contributes to muscle atrophy in chronic kidney disease patients. Redox Rep. 2015;20:126–132. 10.1179/1351000214Y.0000000114 25391884 PMC6837381

[7] Neirynck N, Glorieux G, Schepers E, Verbeke F, Vanholder R. Soluble tumor necrosis factor receptor 1 and 2 predict outcomes in advanced chronic kidney disease: a prospective cohort study. PloS One. 2015;10(3):1–12. 10.1371/journal.pone.0122073 PMC4379033 25823004

[8] Rusu CC, Racasan S, Kacso IM, Moldovan D, Potra A, Patiu IM, et al Malondialdehyde can predict survival in hemodialysis patients. Clujul Med. 2016;89:250 10.15386/cjmed-537 27152077 PMC4849384

[9] Figueiredo PHS, Lima MMO, Costa HS, Gomes RT, Neves CDC, Oliveira ES, et al The role of the inspiratory muscle weakness in functional capacity in hemodialysis patients. PloS One. 2017;12(3): e0173159 10.1371/journal.pone.0173159 28278163 PMC5344350

[10] de Medeiros AIC, Fuzari HKB, Rattesa C, Brandão DC, de Melo Marinho PE. Inspiratory muscle training improves respiratory muscle strength, functional capacity and quality of life in patients with chronic kidney disease: a systematic review. J Physiother. 2017;63(2):76–83 10.1016/j.jphys.2017.02.016 28433237

[11] Montemezzo D, Fregonezi GA, Pereira DA, Britto RR, Reid WD. Influence of inspiratory muscle weakness on inspiratory muscle training responses in chronic heart failure patients: a systematic review and meta-analysis. Arch Phys Med Rehabil. 2014;95:1398–1407. 10.1016/j.apmr.2014.02.022 24631801

[12] Figueiredo RR, Castro AA, Napoleone FMG, Faray L, de Paula Junior AR, Osório RA. Respiratory biofeedback accuracy in chronic renal failure patients: a method comparison. Clin Rehabil. 2012;26:724–732. 10.1177/0269215511431088 22257505

[13] Pellizzaro CO, Thomé FS, Veronese FV. Effect of peripheral and respiratory muscle training on the functional capacity of hemodialysis patients. Ren Fail. 2013;35:189–197. 10.3109/0886022X.2012.745727 23199095

[14] Silva VG, Amaral C, Monteiro MB, Nascimento DM, Boschetti JR. Effects of inspiratory muscle training in hemodialysis patients. J Bras Nefrol. 2011;33:62–68. 21541465

[15] Weiner P, Ganem R, Zamir D, Zonder H. Specific inspiratory muscle training in chronic hemodialysis. Harefuah. 1996;130:73–76. 8846980

[16] Jakel J, Katz D, Elmore J. Epidemiologia, bioestatística e medicina preventiva. 2ª ed Porto Alegre: Artmed 2005.

[17] Smart NA, Williams AD, Levinger I, Selig S, Howden E, Coombes JS, et al Exercise & Sports Science Australia (ESSA) position statement on exercise and chronic kidney disease. J Sci Med Sport. 2013;16:406–411. 10.1016/j.jsams.2013.01.005 23434075

[18] American Thorax Society. ATS/ERS Statement on respiratory muscle testing. Am J Respir Crit Care Med. 2002;166:518–624. 10.1164/rccm.166.4.518 12186831

[19] Neder JA, Andreoni S, Lerario M, Nery L. Reference values for lung function tests: II. Maximal respiratory pressures and voluntary ventilation. Braz J Med Biol Res. 1999;32:719–727. 10412550 10.1590/s0100-879x1999000600007

[20] Singh SJ, Morgan M, Scott S, Walters D, Hardman AE. Development of a shuttle walking test of disability in patients with chronic airways obstruction. Thorax. 1992;47:1019–1024. 1494764 10.1136/thx.47.12.1019 PMC1021093

[21] Probst VS, Hernandes NA, Teixeira DC, Felcar JM, Mesquita RB, Gonçalves CG, et al Reference values for the incremental shuttle walking test. Resp Med. 2012;106:243–248. 10.1016/j.rmed.2011.07.023 21865021

[22] Alves R, Lima M, Fonseca C, Dos Reis R, Figueiredo P, Costa H, et al Peak oxygen uptake during the incremental shuttle walk test in a predominantly female population with Chagas heart disease. Eur J Phys Rehabil Med. 2016;52:20–27. 26530211

[23] da Cunha-Filho IT, Pereira DAG, de Carvalho AMB, Campedeli L, Soares M, de Souza Freitas J. The reliability of walking tests in people with claudication. Am J Phys Med Rehabil. 2007;86:574–582. 10.1097/PHM.0b013e31806de721 17581292

[24] Dourado VZ, Guerra RLF, Tanni SE, Antunes LC, Godoy I. Reference values for the incremental shuttle walk test in healthy subjects: from the walk distance to physiological responses. J Bras Pneumol. 2013;39:190–197. 10.1590/S1806-37132013000200010 23670504 PMC4075833

[25] Csuka M, McCarty DJ. Simple method for measurement of lower extremity muscle strength. The American journal of medicine. 1985;78:77–81. 10.1016/0002-9343(85)90465-6 3966492

[26] Glass GA, Gershon D. Enzymatic changes in rat erythrocytes with increasing cell and donor age: loss of superoxide dismutase activity associated with increases in catalytically defective forms. Biochem Biophys Res Commun. 1981;103:1245–1253. 7332591 10.1016/0006-291x(81)90256-4

[27] Bradford MM. A rapid and sensitive method for the quantitation of microgram quantities of protein utilizing the principle of protein-dye binding. Anal Biochem. 1976;72: 248–254. 942051 10.1016/0003-2697(76)90527-3

[28] Ohkawa H, Ohishi N, Yagi K. Assay for lipid peroxides in animal tissues by thiobarbituric acid reaction. Anal Biochem. 1979;95:351–358. 36810 10.1016/0003-2697(79)90738-3

[29] Benzie IF, Strain J. The ferric reducing ability of plasma (FRAP) as a measure of “antioxidant power”: the FRAP assay. Anal Biochem. 1996;239:70–76. 10.1006/abio.1996.0292 8660627

[30] Srivastava S, Chandrasekar B, Gu Y, Luo J, Hamid T, Hill BG, et al Downregulation of CuZn-superoxide dismutase contributes to β-adrenergic receptor-mediated oxidative stress in the heart. Cardiovascular research. 2007;74:445–455. 10.1016/j.cardiores.2007.02.016 17362897

[31] Nelson DP, Kiesow LA. Enthalpy of decomposition of hydrogen peroxide by catalase at 25C (with molar extinction coefficients of H2O2 solutions in the UV). Anal Biochem. 1972;49(2):474–8. 5082943 10.1016/0003-2697(72)90451-4

[32] Duarte PS, Ciconelli RM, Sesso R. Cultural adaptation and validation of the" Kidney Disease and Quality of Life-Short Form (KDQOL-SF™ 1.3)" in Brazil. Braz J Med Biol Res. 2005;38:261–270. 15785838 10.1590/s0100-879x2005000200015

[33] Jackson MJ. Redox regulation of muscle adaptations to contractile activity and aging. J Appl Physiol. 2015;119:163–171. 10.1152/japplphysiol.00760.2014 25792715 PMC4526708

[34] Siri WE. Body composition from fluid spaces and density: analysis of methods. Techniques for measuring body composition. 1961;61:223–244. 8286893

[35] Gosselink R, De Vos J, Van den Heuvel S, Segers J, Decramer M, Kwakkel G. Impact of inspiratory muscle training in patients with COPD: what is the evidence? Eur Respir J. 2011;37: 416–425. 10.1183/09031936.00031810 21282809

[36] Houchen-Wolloff L, Boyce S, Singh S. The minimum clinically important improvement in the incremental shuttle walk test following cardiac rehabilitation. Eur J Prev Cardiol. 2015;22:972–978. 10.1177/2047487314540840 24958737

[37] Wilkinson TJ, Xenophontos S, Gould DW, Vogt BP, Viana JL, Smith AC, et al Test–retest reliability, validation, and “minimal detectable change” scores for frequently reported tests of objective physical function in patients with non-dialysis chronic kidney disease. Physiother Theory Pract. 2018;30:1–12. 10.1080/09593985.2018.1455249 29601230

[38] Thompson S, Klarenbach S, Molzahn A, Lloyd A, Gabrys I, Haykowsky M, et al Randomised factorial mixed method pilot study of aerobic and resistance exercise in haemodialysis patients: DIALY-SIZE! BMJ. 2016;6(9):e012085. 10.1136/bmjopen-2016-012085 PMC5020875 27601500

[39] Song WJ, Sohng KY. Effects of progressive resistance training on body composition, physical fitness and quality of life of patients on hemodialysis. J Korean Acad Nurs. 2012;42(7):947–56. 10.4040/jkan.2012.42.7.947 23377590

[40] Viana JL, Kosmadakis GC, Watson EL, Bevington A, Feehally J, Bishop NC, et al Evidence for anti-inflammatory effects of exercise in CKD. J Am Soc Nephrol. 2014;25:2121–2130. 10.1681/ASN.2013070702 24700875 PMC4147973

[41] Kato A, Odamaki M, Takita T, Furuhashi M, Maruyama Y, Hishida A. High blood soluble receptor p80 for tumour necrosis factor‐α is associated with erythropoietin resistance in haemodialysis patients. Nephrol Dial Transplant. 2001;16:1838–1844. 11522867 10.1093/ndt/16.9.1838

[42] Wang ZV, Scherer PE. Adiponectin, the past two decades. J Mol Cell Biol. 2016;8:93–100. 10.1093/jmcb/mjw011 26993047 PMC4816148

[43] Bo S, Gambino R, Pagani A, Guidi S, Gentile L, Cassader M, et al Relationships between human serum resistin, inflammatory markers and insulin resistance. Int J Obes. 2005;29:1315–1320. 10.1038/sj.ijo.0803037 16044175

[44] Rashidlamir A, Saadatnia A. The effect of eight weeks of aerobic training on the plasma level of adiponectin, leptin, and resistin in healthy middle-aged men. Science & Sports. 2012;27:351–356.

[45] Perseghin G, Burska A, Lattuada G, Alberti G, Costantino F, Ragogna F, et al Increased serum resistin in elite endurance athletes with high insulin sensitivity. Diabetologia. 2006;49:1893–1900. 10.1007/s00125-006-0267-7 16685503

[46] Boström EA, Svensson M, Andersson S, Jonsson IM, Ekwall AK, Eisler T, et al Resistin and insulin/insulin-like growth factor signaling in rheumatoid arthritis. Arthritis Rheum. 2011;63:2894–2904. 10.1002/art.30527 21739426

[47] Chung W, Jung ES, Shin D, Choi SH, Jung JY, Chang JH, et al Low resistin level is associated with poor hospitalization-free survival in hemodialysis patients. J Korean Med Sci. 2012;27:377–381. 10.3346/jkms.2012.27.4.377 22468100 PMC3314849

[48] Sallam N, Laher I. Exercise modulates oxidative stress and inflammation in aging and cardiovascular diseases. Oxid Med Cell Longev. 2016;2016:1–32. 10.1155/2016/7239639 PMC4707375 26823952

[49] Wilund KR, Tomayko EJ, Wu P-T, Chung HR, Vallurupalli S, Lakshminarayanan B, et al Intradialytic exercise training reduces oxidative stress and epicardial fat: a pilot study. Nephrol Dial Transplant. 2010;25:2695–2701. 10.1093/ndt/gfq106 20190243

[50] Belinda S, Francesco M-R, Eric S, Patrizia P, Sebastiano C, D’Arrigo G, et al Resistin and all-cause and cardiovascular mortality: effect modification by adiponectin in end-stage kidney disease patients. Nephrol Dial Transplant. 2013;28(suppl4):181–7. 10.1093/ndt/gft365 23975745

[51] Ohashi N, Kato A, Misaki T, Sakakima M, Fujigaki Y, Yamamoto T, et al Association of serum adiponectin levels with all-cause mortality in hemodialysis patients. Intern Med. 2008;47:485–491. 18344634 10.2169/internalmedicine.47.0614

[52] Chung YC, Yeh ML, Liu YM. Effects of Intradialytic Exercise on the Physical Function, Depression, and Quality of Life for Hemodialysis Patients: A Systematic Review and Meta-analysis of Randomized Controlled Trials. J Clin Nurs. 2016;26(13–14):1801–1813 10.1111/jocn.13514 27532211

[53] Mandoorah QM, Shaheen FA, Mandoorah SM, Bawazir SA, Alshohaib SS. Impact of demographic and comorbid conditions on quality of life of hemodialysis patients: a cross-sectional study. Saudi J Kidney Dis Transpl. 2014;25:432–7. 24626022 10.4103/1319-2442.128613

