# Supplementary material for: Effects of the inspiratory muscle training and aerobic training on respiratory and functional parameters, inflammatory biomarkers, redox status and quality of life in hemodialysis patients: A randomized clinical trial
Source: PLoS One. 2018 Jul 26;13(7):e0200727. doi: 10.1371/journal.pone.0200727 (PMC6061993; doi:10.1371/journal.pone.0200727)
Supplement: S1 Table — (DOCX) [file pone.0200727.s001.docx]

| **S1 Table. Effects of the interventions on health quality of life** | | | | | | | | | | | | | | | | | | | |
| --- | --- | --- | --- | --- | --- | --- | --- | --- | --- | --- | --- | --- | --- | --- | --- | --- | --- | --- | --- |
| **Variables** | **IMT (n = 10)** | | | | | | | | | | | **AT (n = 10)** | | **CT (n = 11)** | **Within-group** | | | **Between-group** | **interaction** |
|  | | | | | | | |  | | | |  | |  | **p** | **power** | **ES** | **p** | **p** |
| **ESRD component** | | | | | | | |  | | | |  | |  |  |  |  |  |  |
| **Syntoms** | | | | | | | |  | | | |  | |  | 0.13 | 0.24 | --- | 0.91 | 0.77 |
| Baseline | 89.2 (80.6 – 97.7) | | | | | | | | | | | 90.6 (84.5 – 96.8) | | 88.4 (79.8 – 97.1) |  |  |  |  |  |
| 8-week | 88.8 (75.4 – 102.1) | | | | | | | | | | | 89.8 (81.9 – 97.7) | | 85.6 (75.7 – 98.5) |  |  |  |  |  |
| 16-week | 94.8 (90.0 – 99.6) | | | | | | | | | | | 93.3 (88.1 – 98.6) | | 94.4 (87.7 – 97.1) |  |  |  |  |  |
| **Efects of ESRD** | | |  | | | | | | | | |  | |  | 0.44 | 0.05 | --- | 0.42 | 0.49 |
| Baseline | 82.8 (69.9 – 95.8) | | | | | | | | | | | 80.3 (67.8 – 92.8) | | 75.6 (58.0 – 93.2) |  |  |  |  |  |
| 8-week | 80.9 (64.0 – 97.9) | | | | | | | | | | | 79.7 (70.0 – 89.4) | | 79.6 (59.8 – 99.3) |  |  |  |  |  |
| 16-week | 83.6 (71.4 – 95.8) | | | | | | | | | | | 87.0 (74.1 – 99.8) | | 79.3 (64.1 – 94.5) |  |  |  |  |  |
| **Burden of ESRD** | | | | |  | | | | | | |  | |  | 0.10 | 0.30 | --- | 0.62 | 0.62 |
| Baseline | 52.5 (25.8 – 79.2) | | | | | | | | | | | 55.6 (33.7 – 78.0) | | 37.5 (13.8 – 61.2) |  |  |  |  |  |
| 8-week | 56.3 (27.7 – 84.4) | | | | | | | | | | | 61.9 (43.1 – 80.7) | | 45.5 (19.6 – 71.3) |  |  |  |  |  |
| 16-week | 52.4 (21.6 – 83.1) | | | | | | | | | | | 67.7 (37.7 – 97.7) | | 55.7 (32.8 – 78.6) |  |  |  |  |  |
| **Work status** | | | | | | |  | | | | |  | |  | 0.34 | 0.07 | --- | 0.3 | 0.71 |
| Baseline | 45.0 (13.7 – 76.3) | | | | | | | | | | | 40.0 (7.1 – 72.9) | | 13.6 (-8.1 – 35.4) |  |  |  |  |  |
| 8-week | 35.0 (5.5 – 64.5) | | | | | | | | | | | 30.0 (0.2 – 60.2) | | 9.1 (-4.5 – 22.7) |  |  |  |  |  |
| 16-week | 37.5 (0.5 – 74.6) | | | | | | | | | | | 25.0 (-18.9 – 68.9) | | 22.7 (-4.8 – 50.3) |  |  |  |  |  |
| **Cognitive function** | | | | | |  | | | | | |  | |  | 0.10 | 0.29 | --- | 0.7 | 0.83 |
| Baseline | 97.2 (92.5 – 101.9) | | | | | | | | | | | 93.3 (85.9 – 100.8) | | 87.7 (75.0 – 100.5) |  |  |  |  |  |
| 8-week | 90.7 (69.6 – 111.8) | | | | | | | | | | | 92.0 (75.5 – 108.5) | | 90.3 (78.7 – 101.9) |  |  |  |  |  |
| 16-week | 99.2 (97.2 – 101.3) | | | | | | | | | | | 97.8 (92.1 – 103.5) | | 96.4 (90.9 – 101.8) |  |  |  |  |  |
| **Quality of social interaction** | | | | | | | | | | | |  |  |  | 0.30 | 0.08 | --- | 0.5 | 0.77 |
| Baseline | 89.3 (68.3 – 110.3) | | | | | | | | | | | 94.7 (87.0 – 102.4) | | 82.9 (66.1 – 99.6) |  |  |  |  |  |
| 8-week | 94.0 (86.7 – 101.3) | | | | | | | | | | | 90.0 (78.3 – 101.7) | | 88.9 (74.2 – 103.7) |  |  |  |  |  |
| 16-week | 96.7 (88.8 – 104.6) | | | | | | | | | | | 95.6 (84.1 – 107.0) | | 95.2 (77.1 – 103.1) |  |  |  |  |  |
| **Sexual function** | | | | | | | | | | | |  |  |  | 0.15 | 0.21 | --- | 0.18 | 0.46 |
| Baseline | 46.3 (10.5 – 82.0) | | | | | | | | | | | 70.0 (35.5 – 104.6) | | 45.5 (10.4 – 80.5) |  |  |  |  |  |
| 8-week | 58.8 (22.5 – 95.0) | | | | | | | | | | | 56.3 (20.7 – 91.8) | | 50.0 (16.4 – 83.6) |  |  |  |  |  |
| 16-week | 62.5 (19.2 – 105.8) | | | | | | | | | | | 83.3 (40.5 – 126.2) | | 63.6 (32.3 – 94.9) |  |  |  |  |  |
| **Sleep** | | | |  | | | | | | | |  | |  | 0.38 | 0.05 | --- | 0.88 | 0.57 |
| Baseline | 78.8 (60.1 – 97.4) | | | | | | | | | | | 82.8 (66.8 – 98.8) | | 85.0 (72.7 – 97.3) |  |  |  |  |  |
| 8-week | 84.0 (66.7 – 101.5) | | | | | | | | | | | 88.0 (74.4 – 101.6) | | 78.1 (57.2 – 98.9) |  |  |  |  |  |
| 16-week | 80.6 (59.5 – 101.8) | | | | | | | | | | | 95.0 (92.1 – 97.9) | | 86.0 (70.8 – 101.2) |  |  |  |  |  |
| **Social support** |  | | | | | | | | | | |  | |  | 0.14 | 0.21 | --- | 0.58 | 0.27 |
| Baseline | 91.7 (76.5 – 100.0) | | | | | | | | | | | 85.0 (62.9 – 107.1) | | 93.9 (88.3 – 99.6) |  |  |  |  |  |
| 8-week | 80.0 (44.6 – 105.4) | | | | | | | | | | | 81.7 (62.7 – 100.7) | | 92.4 (78.8 – 106.0) |  |  |  |  |  |
| 16-week | 95.0 (85.2 – 104.8) | | | | | | | | | | | 85.6 (38.6 – 122.6) | | 97.7 (79.4 – 106.1) |  |  |  |  |  |
| **Dialysis staff encouragement** | | | | | | | | | | |  |  | |  | 0.79 | 0.05 | --- | 0.80 | 0.49 |
| Baseline | 65.0 (31.1 – 99.0) | | | | | | | | | | | 87.5 (64.8 – 110.2) | | 77.3 (54.2 – 100.4) |  |  |  |  |  |
| 8-week | 75.0 (49.7 – 100.3) | | | | | | | | | | | 75.0 (44.6 – 105.4) | | 83.4 (64.4 – 108.1) |  |  |  |  |  |
| 16-week | 93.8 (79.0 – 108.5) | | | | | | | | | | | 87.5 (67.6 – 107.2) | | 77.3 (54.2 – 100.4) |  |  |  |  |  |
| **Patient satisfaction** | |  | | | | | | | | | |  | |  | 0.58 | 0.05 | --- | 0.51 | 0.78 |
| Baseline | 86.7 (80.0 – 102.4) | | | | | | | | | | | 78.3 (63.4 – 93.3) | | 90.9 (81.7 – 100.1) |  |  |  |  |  |
| 8-week | 85.0 (69.7 – 100.3) | | | | | | | | | | | 78.7 (59.9 – 97.4) | | 79.1 (64.8 – 93.4) |  |  |  |  |  |
| 16-week | 85.4 (68.0 – 102.8) | | | | | | | | | | | 91.7 (77.0 – 106.3) | | 86.4 (75.4 – 97.4) |  |  |  |  |  |
| **SF-36 component** | | | | | | | | | |  | |  | |  |  |  |  |  |  |
| **Physical funtioning** | | | | | | | | | |  | |  | |  | <0.01 | 0.87 | 0.28 | 0.92 | 0.99 |
| Baseline | 81.5 (65.9 – 97.1) | | | | | | | | | | | 77.5 (59.1 – 95.9) | | 80.9 (67.6 – 94.2) |  |  |  |  |  |
| 8-week | 81.0 (68.1 – 93.9) | | | | | | | | | | | 78.5 (59.5 – 97.5) | | 79.6 (64.4 – 94.7) |  |  |  |  |  |
| 16-week | 87.5 (78.6 – 96.4) | | | | | | | | | | | 90.0 (71.5 – 108.5) | | 89.1 (74.2 – 104.0) |  |  |  |  |  |
| **Role Physical** |  | | | | | | | | | | |  | |  | <0.01 | 0.80 | 0.24 | 0.89 | 0.92 |
| Baseline | 75.0 (52.7 – 97.3) | | | | | | | | | | | 77.5 (57.8 – 97.2) | | 72.7 (56.9 – 88.6) |  |  |  |  |  |
| 8-week | 67.5 (39.5 – 95.5) | | | | | | | | | | | 77.5 (53.0 – 102.0) | | 75.0 (56.6 – 93.4) |  |  |  |  |  |
| 16-week | 87.5 (76.3 – 95.5) | | | | | | | | | | | 87.5 (73.1 – 101.9) | | 88.6 (77.1 – 93.4) |  |  |  |  |  |
| **Pain** |  | | | | | | | | | | |  | |  | 0.01 | 0.65 | 0.19 | 0.98 | 0.28 |
| Baseline | 71.8 (52.5 – 91.5) | | | | | | | | | | | 74.5 (53.0 – 96.0) | | 74.6 (56.8 – 92.3) |  |  |  |  |  |
| 8-week | 89.7 (79.2 – 100.3)^a^ | | | | | | | | | | | 78.0 (58.4 – 97.6) | | 79.3 (59.3 – 99.4) |  |  |  |  |  |
| 16-week | 84.7 (68.6 – 100.7) | | | | | | | | | | | 86.3 (72.8 – 99.7) | | 99.1 (75.3 – 107.0) |  |  |  |  |  |
| **General health** |  | | | | | | | | | | |  | |  | 0.12 | 0.25 | --- | 0.40 | 0.06 |
| Baseline | 80.0 (64.9 – 95.1) | | | | | | | | | | | 47.5 (32.3 – 62.7) | | 60.5 (40.5 – 80.4) |  |  |  |  |  |
| 8-week | 71.0 (52.8 – 89.2) | | | | | | | | | | | 58.5 (44.1 – 72.9) | | 58.2 (36.7 – 79.7) |  |  |  |  |  |
| 16-week | 66.3 (48.6 – 83.9) | | | | | | | | | | | 76.7 (48.2 – 105.2) | | 75.0 (52.8 – 97.2) |  |  |  |  |  |
| **Emotional being health** | | | | | | | | |  | | |  | |  | 0.02 | 0.36 | 0.14 | 0.27 | 0.97 |
| Baseline | 76.7 (54.0 – 99.3) | | | | | | | | | | | 73.3 (41.9 – 100.0) | | 51.5 (22.6 – 80.5) |  |  |  |  |  |
| 8-week | 66.7 (63.6 – 109.7) | | | | | | | | | | | 83.3 (57.6 – 100.0) | | 69.7 (40.6 – 98.8) |  |  |  |  |  |
| 16-week | 91.7 (72.0 – 111.4) | | | | | | | | | | | 87.5 (70.3 – 104.7) | | 78.8 (53.7 – 103.9) |  |  |  |  |  |
| **Role emotional** |  | | | | | | | | | | |  | |  | 0.08 | 0.31 | --- | 0.48 | 0.86 |
| Baseline | 88.4 (74.9 – 101.9) | | | | | | | | | | | 89.2 (73.5 – 104.9) | | 77.5 (57.3 – 97.6) |  |  |  |  |  |
| 8-week | 85.6 (67.3 – 103.9) | | | | | | | | | | | 82.8 (67.8 – 97.8) | | 74.9 (53.0 – 96.8) |  |  |  |  |  |
| 16-week | 90.0 (78.7 – 101.3) | | | | | | | | | | | 98.7 (95.2 – 102.1) | | 84.4 (72.1 – 97.6) |  |  |  |  |  |
| **Social function** |  | | | | | | | | | | |  | |  | 0.30 | 0.08 | --- | 0.51 | 0.78 |
| Baseline | 97.5 (91.8 – 103.1) | | | | | | | | | | | 91.3 (82.8 – 99.7) | | 80.7 (62.5 – 98.8) |  |  |  |  |  |
| 8-week | 93.7 (82.4 – 105.1) | | | | | | | | | | | 92.5 (80.4 – 104.6) | | 95.5 (89.8 – 101.1) |  |  |  |  |  |
| 16-week | 98.4 (94.5 – 102.1) | | | | | | | | | | | 89.6 (74.2 – 104.9) | | 97.7 (92.7 – 102.8) |  |  |  |  |  |
| **Energy/fatigue** |  | | | | | | | | | | |  | |  | 0.01 | 0.70 | 0.28 | 0.25 | 0.54 |
| Baseline | 85.8 (75.2 – 96.4) | | | | | | | | | | | 81.5 (66.7 – 96.6) | | 67.7 (47.3 – 88.2) |  |  |  |  |  |
| 8-week | 86.0 (73.4 – 98.6) | | | | | | | | | | | 84.5 (73.9 – 95.1) | | 73.2 (51.8 – 94.6) |  |  |  |  |  |
| 16-week | 93.8 (86.4 – 101.1) | | | | | | | | | | | 92.3 (82.5 – 102.1) | | 90.0 (79.6 – 97.1)^a.b^ |  |  |  |  |  |

Data represented as mean (IC 95%). IMT: inspiratory muscle training group; AT: aerobic training group; CT: combined training group; ESRD: end-stage renal disease; ES: effect size; Baseline to 8-week: control period; 8-week to 16-week: intervention period. a: p<0.05 to baseline; b: p<0.05 to 8-week.
