# Supplementary material for: Effects of the inspiratory muscle training and aerobic training on respiratory and functional parameters, inflammatory biomarkers, redox status and quality of life in hemodialysis patients: A randomized clinical trial
Source: PLoS One. 2018 Jul 26;13(7):e0200727. doi: 10.1371/journal.pone.0200727 (PMC6061993; doi:10.1371/journal.pone.0200727)
Supplement: S2 File — (PDF) [file pone.0200727.s003.pdf]

STUDY PROTOCOL (Summary from the Brazilian original protocol)

Study: EFFECTS OF THE INSPIRATORY MUSCLE TRAINING AND AEROBIC TRAINING ON RESPIRATORY AND FUNCTIONAL PARAMETERS, INFLAMMATORY BIOMARKERS, REDOX STATUS AND QUALITY OF LIFE IN HEMODIALYSIS PATIENTS: A RANDOMIZED CLINICAL TRIAL

CAAE: 37412314.5.0000.5098

Principal investigator: Pedro Henrique Scheidt Figueiredo[1,2]

Other investigators:

Márcia Maria Oliveira Lima[2], Henrique Silveira Costa[3], Jeanne Branda Martins[1], Olga Dumont Flecha[4], Patrícia Furtado Gonçalves[4], Frederico Lopes Alves[5,6], Vanessa Gomes Brandão Rodrigues[5,6], Emílio Henrique Barroso Maciel[5,6], Vanessa Amaral Mendonça[1,2], Ana Cristina Rodrigues Lacerda [1,2], Érica Leandro Marciano Vieira[3], Antonio Lúcio Teixeira[3], Fabricio de Paula[5], Cláudio Heitor Balthazar [1,2].

1. Programa Multicêntrico de Pós-Graduação em Ciências Fisiológicas-PMPGCF, Universidade Federal dos Vales do Jequitinhonha e Mucuri (UFVJM), Diamantina, Minas Gerais, Brazil
2. Physical Therapy School, Universidade Federal dos Vales do Jequitinhonha e Mucuri (UFVJM), Diamantina, Minas Gerais, Brazil.
3. Medical School, Universidade Federal de Minas Gerais (UFMG), Belo Horizonte, Minas Gerais, Brazil
4. Dentistry School, Universidade Federal dos Vales do Jequitinhonha e Mucuri, Diamantina, Minas Gerais, Brazil
5. Hemodialysis Unit of the Santa Casa de Caridade de Diamantina Hospital, Diamantina, Minas Gerais, Brazil.
6. Medical School, Universidade Federal dos Vales do Jequitinhonha e Mucuri (UFVJM), Diamantina, Minas Gerais, Brazil.

Corresponding author:
Pedro Henrique Scheidt Figueiredo
phsfig@yahoo.com.br (PHSF)

OBJECTIVE

Evaluate and compare the isolated and combined effects of the inspiratory muscle training (IMT) and low intensity aerobic training (AT) on nutritional, respiratory and functional parameters, inflammatory biomarkers and redox status, as well as on the health-related quality of life (HRQoL) domains of hemodialysis patients.

HYPOTHESES

The alternative hypothesis this study will be that the improvements induced by IMT are lower than that induced by low intensity AT and that the combined IMT and TA induce greater improvements than the isolated approaches.

METHODS

Sample selection

Inclusion criteria

- Subjects with chronic kidney disease on hemodialisys treatment for three times a week for at least three months;

- Had to be more than 18 years old;

- Arteriovenous fistula for hemodialysis access;

- Non-users of anti-inflammatory (hormonal or non-hormonal) or anti-allergic.

Exclusion Criteria

Exclusion criteria will be any contraindication to physical exercise or inability to perform the functional tests

Study design

In this randomised controlled trial with factorial allocation and intention-to-treat analysis, participants will be randomly allocated into three experimental condictions: IMT, AT or combined training (CT). After selection of participants, the volunteers will be stratified by hemodialysis schedule (7:00h, 12:30h e 16:30h). Randomisation will be performed using individual allocation codes placed within opaque, sealed envelopes by a person having no contact with the participants. Potential participants will be screened to verify eligibility before baseline assessment and randomization. The eight weeks prior to initiating the interventions will be considered to represent the control period (Fig. 1).

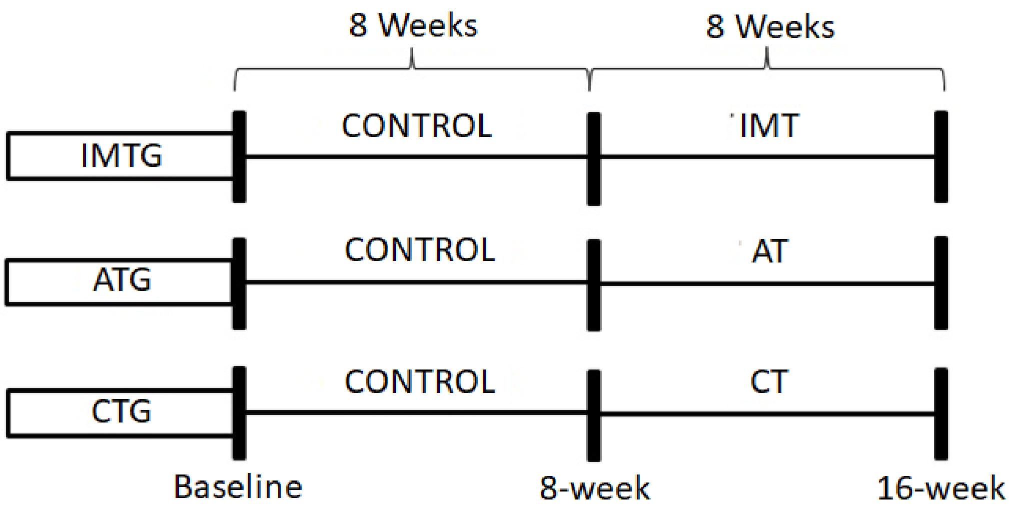

Figure 1. Study design. IMT: inspiratory muscle training; AT: low intensity aerobic training; CT: combined training; IMTG: IMT group; ATG: AT group; CTG: CT group.

Measurement instruments and procedures

After medical and dental evaluation, the volunteers will be evaluated in the baseline, 8-week follow-up (after control period) and 16-week follow-up (after intervention period), on dialitic days, immediately before the hemodialysis sessions, except for the evaluation of body composition, in the following sequence:

- 1st hemodialysis weekly session: Anamnesis, physical examination and evaluation of respiratory muscle strength and lower limbs strength;

- 2nd hemodialysis weekly session: HRQOL assessment, blood collection and fat percentage assessment;

- 3rd hemodialysis weekly session: functional capacity evaluation;

The evaluations will be performed by properly trained researchers, who did not know the other results, when possible, except the clinical examination. All volunteers will be previously familiar with the evaluation methods.

Heigth, weight and body mass index

The body mass and height will be measured by means of a properly calibrated scale (Líder Balança, LD 1050) and the BMI calculated by the ratio: weight / height^2.

Waist circumference

After recording body mass and height, waist circumference will be measured with the volunteers in an orthostatic position, keeping the hands flat against the lateral sides of the thigh and the lower limbs adducted. Using a tape measure, waist circumference measurement will be performed 2.5 cm above the umbilical scar, after a basal expiration, as recommended by the American College of Sports Medicine.[1]

Body fat percentage

Body fat percentage will be evaluated by an analogic plicometer (Sanny, American Medical do Brasil, Brasil). Three sinkfolds (tricipital, suprailiac and abdominal in womans and tricipital, subscapular and middle pectoral in mens) will be measured on the right side. The percentage of fat mass will be obtained by use of the equations of Jackson & Pollock (1985)[2] and Siri (1961).[3]

Lower limbs strength

It will be performed by Sit-to-Stand test of 30 s. The evaluation will be initiated with the volunteer sitting in a 45 cm high chair with backrest, feet resting on the ground and lower limbs apart and aligned, with the shoulders line as a reference. The volunteers will be instructed to stand up completely with their upper limbs crossed on the trunk, resting their hands on the contralateral shoulder and sitting again, as many times as possible in 30s, without verbal stimulus.[4]

After explanations about the test and demonstration, two tests will be performed, with a minimum interval of five minutes between both or until the vital signs and the sensation of effort, assessment by the modified Borg scale, return to the resting condition. To analysis will be considered the best performing test. The variable of interest will be the number of movements completed in 30s.[4]

Before and after the evaluation vital signs and sensation of exertion (modified Borg scale) will be recorded.

Inspiratory Muscle Strength

Respiratory muscle strength will be determined with a previously calibrated aneroid vacuum manometer (MV-150/300, Ger-Ar, São Paulo, Brazil), equipped with a 2mm

diameter hole in the nozzle to compensate for the pressure change induced by the oropharynx muscles. The Maximal Inspiratory Pressure will be evaluated from residual volume with the volunteers seated. The highest value of three valid measurements will be considered.[5,6] The measures will be considered satisfactory if variance between them are lower 10% at maximum. Respiratory measurements will be show in absolute and relative values by the percentage achieved compared to the maximum predicted by age and sex.[7] Inspiratory muscle weakness will be considered when the MIP will less than 70% of predicted value.[5]

Functional capacity

The functional capacity will be evaluated by the Incremental Shuttle Walk Test (ISWT), according the protocol proposed by Singh et al.[8] Volunteers will be instructed to walk or run in a 10 m corridor and the minimum speed will be determined by an audio signal. The ISWT has 12 progressive intensity levels, and the test will be finished when the volunteer either completed the 12 levels of intensity or failed to reach the minimum speed required on a level two consecutive times.[8] Vital signs will be monitored during the test and the distance walked will be recorded. The values will be presented in absolute and relative values by the percentage achieved compared to the maximum predicted.[9]

Inflamatory Biomarkers and Redox status parameters

Ethylenediaminetetraacetic acid vacutainers (BD vacutainers, Franklin Lakes, NJ, USA) will be used to collect 8-ml venous blood samples immediately before the second weekly hemodialysis session (midweek), without fasting. The tubes will be centrifuged at 2500 rpm for 10 min at 4 ºC and stored as plasma and erythrocyte aliquots at -80 ˚C until use.

Inflammatory biomarkers

Plasma soluble tumor necrosis factor receptor 1 and 2 (sTNFR1 and sTNFR2, respectively), leptin, adiponectin and resistin levels will be measured using conventional sandwich ELISA kits (DuoSet, R&D Systems, Minneapolis, MN, USA) according to the manufacturer's instructions. The plasma interleukin 6 (IL-6) level will be measured using the cytometric bead arrays kit (BD Bioscience, San Jose, CA, USA) according to the manufacturer's protocol. Samples will be acquired in a FACSCanto flow cytometer (BD Biosciences, San Jose, CA, USA) and analyzed using the FCAP Array v1.0.1 software (Soft Flow Inc.).

Redox status parameters

The redox status will be assessed by thiobarbituric acid reactive substances (TBARS) concentration, antioxidants enzymes dismutase superoxide (SOD) and catalase (CAT) activity, as well by ferric reducing antioxidant power (FRAP) in the erythrocyte lysate. The erythrocyte lysate will be prepared as described by Glass and Gershon.[10] Protein concentration of samples will be determined by the Bradford method [11] using bovine serum albumin (BSA) (1 mg/mL) as standard.

TBARS concentration will be measured according to the method described by Ohkawa et al.,[12] and the reaction of the thiobarbituric acid with malondialdehyde (MDA) will be used to determine lipid peroxidation. TBARS concentration, expressed in nmol MDA/mg protein, will be determined from the standard curve constructed with known concentrations of MDA (1,1,3,3-tetramethoxypropane) (Sigma, USA). The assay to determine SOD (EC 1.15.1.1) activity will be performed according to Srivastava et al. [13] and expressed in U/mg of protein. SOD activity will be determined by measuring

the capacity of SOD to inhibit the autoxidation of pyrogallol. CAT (EC 1.11.1.6) activity will be measured according to the method of Nelson and Kiesov [14] and expressed by ΔE/min/mg protein, where ΔE represents the variation in enzyme activity during 1 minute. The total antioxidant capacity (FRAP) will be determined according to the method of Benzie and Strain,[15] which is based on the reduction of ferric-tripyridyltriazine [Fe(III)-TPTZ] complex to ferrous-tripyridyltriazine [Fe(II)-TPTZ]. The total antioxidant capacity will be expressed as equivalents of Fe^{2+}, estimated by comparison with a standard curve constructed with known concentrations of $FeSO_4$ and expressed as μg $FeSO_4$/mg of protein.

Health-related quality of life (HRQoL) **assessment**

The HRQoL will be evaluated by the Brazilian version of the specific questionnaire — KDQOL-SF- Kidney Disease Quality of Life.[16] The KDQOL-SF consist of kidney disease-targeted domains and a health status questionnaire (Short Form-36 [SF-36]). Each category generates a score ranging between 0 and 100, with a higher score indicating a better HRQoL.

Interventions

All interventions (IMT, AT and CT) will be intradialitic, and they will be performed during the first two hours of dialysis, three times a week for eight weeks or 24 sessions.

Inspiratory muscle training

IMT will be performed using Threshold IMT® (Respironics, Murrysville PA, USA) or PowerBreathe light or median Resistance (Powerbreathe, HaB International Ltd, Southam, UK), according to the previously evaluated MIP. Participants performed three

sets of 15 inspirations at the equipment mouthpiece and rested for 60 seconds with the linear load adjusted to 50% of MIP.[17] MIP will be reevaluated every six sessions for load adjustment.

Aerobic training

The AT will be performed by cycle ergometer (Mini Bike E5, ACTE Sports, São Paulo, Brasil) positioned in the front of patients' chairs. The cycling session consisted of a 5-minute warm-up, 30 minutes of cycling at target workload, and a 5-minute cooling-down period. During exercise, patients will be asked every 5 minutes about the fatigue score, and the cycle ergometer load will be adjusted to achieve a fatigue score between three and five points in the modified Borg Scale. The speed remained ≥ 50 rpm.[18] Blood pressure will be monitored at rest, every 5 minutes of conditioning and after cooling. The HR will be continuously monitored by means of a heart rate monitor (Polar F1, Polar ElectroOy, Kempele, Finland) and recorded data every 5 minutes.

Combined training

In the CT sessions, IMT will be performed immediately before AT and, in the AT group, the participants performed sets of inspirations with IMT devices, but without resistance to inspiration (SHAM-IMT).

Control period

The eight weeks prior to initiating the interventions will be considered to represent the control period. During eight weeks prior to initiating the interventions the volunteers did not receive any type of training to represent the control period. The dialysis prescription and medication therapy remained unchanged during the study.

Statistical analysis

The Sigma Stat (Version 9.0) statistic program will be used for the statistical analysis. The data distributions will be verified using the Shapiro-Wilk test. Categorical variables will be presented as absolute and relative frequencies, and continuous variables as the mean (IC95%). Categorical variables will be compered by the Chi-squared test. The effects of the interventions will be compered by Two-Way Repeated Measures ANOVA (within-group, between-group and interactions) with Tukey's post hoc test. For variables with a statistical significance to the within-group differences, the comparisons of the 8-week to 16-week variations (intervention period) will be performed by the one-way ANOVA or Kruskall Wallis test, with Tukey's post hoc test. The significance level will be set at 0.05.

Sample size

The sample size was calculated, considering functional capacity variations of 47% between IMT and lower limb training, standard deviation of 30%,[17] power of 85% and alpha of 5%. The sample size was estimated in 10 volunteers per group.

REFERENCES

1. Whaley M, Brubaker P, Otto R (2007) Diretrizes do ACSM para os testes de esforço e sua prescrição. Diretrizes do ACSM para os testes de esforço e sua prescrição.
2. Jackson (2015) Redox regulation of muscle adaptations to contractile activity and aging. Journal of applied physiology 119: 163-171.
3. Siri WE (1961) Body composition from fluid spaces and density: analysis of methods. Techniques for measuring body composition 61: 223-244.
4. Song W-J, Sohng K-Y (2012) Effects of progressive resistance training on body composition, physical fitness and quality of life of patients on hemodialysis. Journal of Korean Academy of Nursing 42: 947-956.
5. Society AT (2002) ATS/ERS Statement on respiratory muscle testing. Am J Respir Crit Care Med 166: 518-624.
6. Souza RB (2002) Brazilian Society of Pneumology and Tisiology: Guidelines for pulmonary function tests. Maximal static respiratory pressures. J pneumol 28: S1-S238.
7. Neder JA, Andreoni S, Lerario M, Nery L (1999) Reference values for lung function tests: II. Maximal respiratory pressures and voluntary ventilation. Brazilian journal of medical and biological research 32: 719-727.

8. Singh SJ, Morgan M, Scott S, Walters D, Hardman AE (1992) Development of a shuttle walking test of disability in patients with chronic airways obstruction. Thorax 47: 1019-1024.
9. Dourado VZ, Guerra RLF, Tanni SE, Antunes LCdO, Godoy I (2013) Reference values for the incremental shuttle walk test in healthy subjects: from the walk distance to physiological responses. Jornal Brasileiro de Pneumologia 39: 190-197.
10. Glass GA, Gershon D (1981) Enzymatic changes in rat erythrocytes with increasing cell and donor age: loss of superoxide dismutase activity associated with increases in catalytically defective forms. Biochemical and biophysical research communications 103: 1245-1253.
11. Bradford MM (1976) A rapid and sensitive method for the quantitation of microgram quantities of protein utilizing the principle of protein-dye binding. Analytical biochemistry 72: 248-254.
12. Ohkawa H, Ohishi N, Yagi K (1979) Assay for lipid peroxides in animal tissues by thiobarbituric acid reaction. Analytical biochemistry 95: 351-358.
13. Srivastava S, Chandrasekar B, Gu Y, Luo J, Hamid T, et al. (2007) Downregulation of CuZn-superoxide dismutase contributes to β-adrenergic receptor-mediated oxidative stress in the heart. Cardiovascular research 74: 445-455.
14. Nelson DP, Kiesow LA (1972) Enthalpy of decomposition of hydrogen peroxide by catalase at 25C (with molar extinction coefficients of H2O2 solutions in the UV). DTIC Document.
15. Benzie IF, Strain J (1996) The ferric reducing ability of plasma (FRAP) as a measure of “antioxidant power”: the FRAP assay. Analytical biochemistry 239: 70-76.
16. Duarte PS, Ciconelli RM, Sesso R (2005) Cultural adaptation and validation of the" Kidney Disease and Quality of Life-Short Form (KDQOL-SF™ 1.3)" in Brazil. Brazilian journal of medical and biological research 38: 261-270.
17. Pellizzaro CO, Thomé FS, Veronese FV (2013) Effect of peripheral and respiratory muscle training on the functional capacity of hemodialysis patients. Renal failure 35: 189-197.
18. Smart, Williams AD, Levinger I, Selig S, Howden E, et al. (2013) Exercise & Sports Science Australia (ESSA) position statement on exercise and chronic kidney disease. Journal of Science and Medicine in Sport 16: 406-411.
