# Supplementary material for: Effects of the inspiratory muscle training and aerobic training on respiratory and functional parameters, inflammatory biomarkers, redox status and quality of life in hemodialysis patients: A randomized clinical trial
Source: PLoS One. 2018 Jul 26;13(7):e0200727. doi: 10.1371/journal.pone.0200727 (PMC6061993; doi:10.1371/journal.pone.0200727)
Supplement: S3 File — (PDF) [file pone.0200727.s004.pdf]

## PARECER CONSUBSTANCIADO DO CEP

### DADOS DO PROJETO DE PESQUISA

**Título da Pesquisa:** COMPARAÇÃO DOS EFEITOS DO TREINAMENTO MUSCULAR INSPIRATÓRIO E DO TREINAMENTO AERÓBIO DOS MEMBROS INFERIORES EM PARÂMETROS FISIOLÓGICOS E FUNCIONAIS DE PACIENTES EM HEMODIÁLISE

**Pesquisador:** PEDRO HENRIQUE SCHEIDT FIGUEIREDO

**Área Temática:**

**Versão:** 2

**CAAE:** 37412314.5.0000.5098

**Instituição Proponente:**

**Patrocinador Principal:** Universidade Federal dos Vales do Jequitinhonha e Mucuri

### DADOS DO PARECER

**Número do Parecer:** 868.686

**Data da Relatoria:** 10/11/2014

**Apresentação do Projeto:**

A doença renal crônica (DRC) pode ser definida como a perda da função do rim, de forma lenta e progressiva, independente da causa, que, em estágios mais avançados, impossibilitam o sistema renal de desenvolver suas funções regulatórias, sendo necessária a terapia de substituição renal (TSR). A hemodiálise (HD) é a modalidade de TSR mais realizada em pacientes com DRC. Apesar de ser um procedimento fundamental para controle dos sintomas e aumentar a sobrevida dos pacientes, a HD está associada ao desencadeamento e manutenção da resposta inflamatória sistêmica e alterações neuroendócrinas, que podem afetar a musculatura esquelética, impactando negativamente na capacidade funcional e na qualidade de vida relacionada à saúde desta população. Um dos grupamentos musculares afetados pela DRC dialítica é a musculatura inspiratória. Neste sentido, o treinamento deste grupamento muscular (TMI) vem sendo citado como uma modalidade de treinamento físico a se considerada nos programas de reabilitação desses indivíduos. Porém, poucos estudos avaliaram o impacto agudo e crônico desta modalidade de treinamento em parâmetros funcionais, inflamatórios e neuroendócrinos desta população e sua possível influência na qualidade de vida relacionada à saúde. Assim, este estudo objetivará avaliar a relação entre fatores inflamatórios e neuroendócrinos com capacidade funcional, força muscular e QVRS de pacientes em hemodiálise, assim como avaliar os efeitos agudos e crônicos do TMI

isolado e associado ao treinamento aeróbio dos membros inferiores em parâmetros inflamatórios, neuroendócrinos e funcionais desse grupo de pacientes. . Para tal, 48 pacientes serão aleatoriamente alocados em quatro grupos: grupo TMI (GTMI); grupo treinamento aeróbio para membros inferiores (GTA); grupo TMI associado ao treinamento aeróbio para membros inferiores (GTC); e grupo controle (GC). Os voluntários do GTMI realizará o TMI com o dispositivo de carga linear pressórica Threshold IMT®, em três séries de 15 repetições a 50% da pressão inspiratória máxima (PImáx), determinada pela manovacuometria. O GTA realizará o treinamento dos membros inferiores por meio de um cicloergômetro portátil, mantendo a cadência de pedala a 50rpm e intensidade do exercício estipulada pela sensação de esforço de três e sete pela escala modificada de Borg, por 30min. Os indivíduos do GTC realizarão o TMI antes do treinamento dos membros inferiores. Todas as sessões de exercícios serão intradialíticas, realizadas nas duas primeiras horas da sessão de HD, três vezes por semana, durante 10 semanas. Os voluntários do GC não sofrerão nenhuma intervenção durante o segmento do estudo. Antes e após a execução das intervenções e do acompanhamento do GC os voluntários serão avaliados quanto a: força dos membros inferiores pelo Teste de Sentar e Levantar de 30s; força muscular inspiratória por meio da mensuração da PImáx, através de um manovacuômetro analógico; qualidade de vida relacionada à saúde pelo questionário específico para DRC dialítica KDQOL-SF; capacidade funcional pela mensuração da distância e medida direta do consumo de oxigênio de pico durante o Shuttle Walk Test; coleta sanguínea para dosagem da concentração de marcadores inflamatórios (IL-6, TNF- e PCR); e coleta de saliva para dosagem da concentração de cortisol salivar. Todas as avaliações ocorrerão em dias dialíticos, imediatamente antes das sessões de HD. Para caracterização e comparação dos efeitos agudos das intervenções na concentração dos marcadores inflamatórios e na concentração de cortisol salivar, os indivíduos terão o sangue e saliva coletados antes e após a sessão de HD em que for aplicada a primeira sessão dos programas de exercícios (GTA, GTMI, GTC) ou do GC.

**Objetivo da Pesquisa:**

Objetivo Primário:

Avaliar a relação entre fatores inflamatórios e neuroendócrinos com capacidade funcional, força muscular e QVRS de pacientes em hemodiálise, assim como avaliar os efeitos agudos e crônicos do TMI isolado e associado ao treinamento aeróbio dos membros inferiores em parâmetros inflamatórios, neuroendócrinos e funcionais desse grupo de pacientes.

Objetivo Secundário:

- Avaliar a capacidade funcional;- Avaliar a qualidade de vida relacionada à saúde;- Avaliar a força

muscular respiratória;- Analisar a concentração sérica de citocinas e de proteína C reativa;- Analisar a concentração e o padrão de variação diária de cortisol salivar;- Avaliar a força muscular esquelética dos membros inferiores;- Avaliar a associações entre essas medidas;- Comparar os efeitos do treinamento aeróbio e do TMI aplicados de forma isolada e associada nas variáveis acima descritas; - Comparar os efeitos agudos do exercício aeróbio e do TMI aplicados de forma isolada e associada na concentração sérica de citocinas e proteína C reativa e de cortisol salivar.

**Avaliação dos Riscos e Benefícios:**

Riscos:

Os riscos associados com a participação do voluntário podem ser desconforto ou constrangimento ao responder a ficha de avaliação e questionários, o que será amenizado ao ser realizado com profissionalismo após treinamento dos pesquisadores e em local privativo. A coleta sanguínea causa dor e desconforto durante a coleta. Entretanto, este procedimento faz parte do acompanhamento clínico e da rotina diária da sessão de HD destes pacientes. Além disso, será realizado por profissional treinado do próprio setor de HD.Os riscos com o teste de esforço submáximo serão minimizados por ser aplicado por pessoal treinado, em ambiente hospitalar, na presença de médico e com suporte de emergência.Os riscos e desconfortos induzidos pelas sessões de exercício serão minimizados visto que a intensidade do treinamento será prescrita de forma individualizada, as sessões de exercício serão supervisionadas por profissional treinado, na presença de médico e com suporte de
emergência.

Benefícios:

Os benefícios associados com a participação do voluntário incluem a execução de exames e avaliações não rotineiras: o conhecimento de dados relacionados a fatores de risco cardiovascular, como inatividade física, índice de massa corporal, medidas e respostas cardiovasculares ao exercício, resposta da frequência cardíaca aos estímulos e outras variáveis coletadas na anamnese, estabelecendo, portanto, uma estratégia preventiva. Todos os voluntários receberão fichas individuais com os resultados da avaliação. No mais, os benefícios do treinamento aeróbico no controle dos fatores de risco cardiovascular, no aumento da sobrevida e na melhora da QVRS dessa população já estão amplamente divulgados na literatura científica. Vale ressaltar que esta modalidade de treinamento está contemplada em dois dos três protocolos de treinamento propostos neste estudo.Entretanto, após análise dos resultados, todos os voluntários passarão pelo programa de treinamento físico que desencadear respostas mais satisfatórias.

**Endereço:** Rua dos Guajajaras, 175
**Bairro:** Centro **CEP:** 30.180-100
**UF:** MG **Município:** BELO HORIZONTE
**Telefone:** (31)3508-9110 **E-mail:** cephumanos@una.br

**Comentários e Considerações sobre a Pesquisa:**

O projeto possui justificativa e relevância. Não há dúvidas quanto a pertinência e valor científico do estudo proposto. Quanto aos aspectos éticos, o projeto evidencia a necessidade do Termo de Livre Consentimento e este se encontra anexado. Registra-se a presença dos compromissos exigidos do pesquisador e da instituição responsável, a garantia dos direitos fundamentais do sujeito de pesquisa (informação, privacidade, recusa inócua, desistência, etc). O modelo de questionário também se encontra em anexo o que permite a análise de seu conteúdo e a verificação dos aspectos éticos. Observa-se ainda que o projeto presenta orçamento viável.

**Considerações sobre os Termos de apresentação obrigatória:**

Apresenta folha de rosto devidamente assinada pela instituição proponente, currículo lattes dos pesquisadores, cronograma de execução, tabela de custos, termo de consentimento livre e esclarecido, roteiro para coleta de dados e autorização para realização da pesquisa.

**Recomendações:**

Ver o item conclusões.

**Conclusões ou Pendências e Lista de Inadequações:**

Foram atendidas as pendências 01) apresentação da carta de anuência da Santa Casa e 02) descrição do processo de acompanhamento dos pacientes. Segundo o pesquisador o procedimento será supervisionado por profissional treinado, na presença de médico e com suporte de emergência. O colegiado vota, portanto, pela aprovação.

**Situação do Parecer:**

Aprovado

**Necessita Apreciação da CONEP:**

Não

**Considerações Finais a critério do CEP:**

BELO HORIZONTE, 12 de Novembro de 2014

**Assinado por:**
**ELAINE LINHARES DE ASSIS GUERRA**
**(Coordenador)**

## RELATÓRIO FINAL DE PROJETO DE PESQUISA

| |
|---|
| Nº CAAE: 37412314.5.0000.5098 |
| TÍTULO DO PROJETO:<br><br>COMPARAÇÃO DOS EFEITOS DO TREINAMENTO MUSCULAR INSPIRATÓRIO E DO TREINAMENTO AERÓBIO DOS MEMBROS INFERIORES EM PARÂMETROS FISIOLÓGICOS E FUNCIONAIS DE PACIENTES EM HEMODIÁLISE |
| PESQUISADOR RESPONSÁVEL: Pedro Henrique Scheidt Figueiredo |
| Local de realização da pesquisa: Santa Casa de Caridade de Diamantina, Diamantina-MG |
| Estágio atual do projeto<br>Data do Fechamento do arrolamento dos sujeitos da pesquisa: 10/12/2015<br>Total de sujeitos arrolados: 43 |
| Resultados já apresentados em congresso X Sim ☐ Não<br>EVENTO : XVIII Simpósio Internacional de Fisioterapia Respiratória e Fisioterapia em Terapia Intensiva. |
| Resultados totais já publicados ☐ Sim X Não<br>REFERÊNCIA : |
| Perspectivas de publicações: Periódico PlosOne, no segundo semestre de 2017 |
| Ocorrência de efeitos adversos ☐ Sim X Não<br>Descrição dos efeitos adversos: |
| Comentários:<br>O projeto foi finalizado sem efeitos adversos aos voluntários da pesquisa. Dos 43 voluntários arrolados, 31 completaram todas as etapas do estudo. O projeto foi planejado para formação de quatro grupos experimentais, sendo três grupos de intervenções e um grupo controle. Devido ao reduzido número de voluntários aptos a participar da pesquisa, o grupo controle foi extinto e todos os voluntários passaram pelo período de controle, que ocorreu imediatamente antes do período de intervenção.<br>As três intervenções aplicadas desencadearam efeitos benéficos similares. Com os resultados do estudo, o exercício físico foi introduzido pelo grupo de pesquisa no setor de hemodiálise em que o trabalho foi realizado e, até a presente data, permanece como uma intervenção clinica da referida unidade. Os sujeitos da pesquisa que optaram por continuar em treinamento físico ainda permanecem praticando atividade física supervisionada. |

| | |
|---|---|
| Data: 18/08/2017 | |
| Assinatura do Pesquisador | [signature] |

# PARECER CONSUBSTANCIADO DO CEP

**DADOS DO PROJETO DE PESQUISA**

**Título da Pesquisa:** COMPARAÇÃO DOS EFEITOS DO TREINAMENTO MUSCULAR INSPIRATÓRIO E DO TREINAMENTO AERÓBIO DOS MEMBROS INFERIORES EM PARÂMETROS FISIOLÓGICOS E FUNCIONAIS DE PACIENTES EM HEMODIÁLISE

**Pesquisador:** PEDRO HENRIQUE SCHEIDT FIGUEIREDO

**Área Temática:**

**Versão:** 2

**CAAE:** 37412314.5.0000.5098

**Instituição Proponente:**

**Patrocinador Principal:** Universidade Federal dos Vales do Jequitinhonha e Mucuri

**DADOS DA NOTIFICAÇÃO**

**Tipo de Notificação:** Envio de Relatório Final

**Detalhe:**

**Justificativa:**

**Data do Envio:** 25/08/2017

**Situação da Notificação:** Parecer Consubstanciado Emitido

**DADOS DO PARECER**

**Número do Parecer:** 2.320.579

**Apresentação da Notificação:**

Relatório final do projeto intitulado "Comparação dos efeitos do treinamento muscular inspiratório e do treinamento aeróbio dos membros inferiores em parâmetros fisiológicos e funcionais de pacientes em hemodialise".

**Objetivo da Notificação:**

Notificar ao CEP a finalização do projeto e as publicações relacionadas ao projetos.

**Avaliação dos Riscos e Benefícios:**

O projeto foi finalizado sem efeitos adversos aos voluntários da pesquisa.

**Endereço:** Rua dos Guajajaras, 175
**Bairro:** Centro **CEP:** 30.180-100
**UF:** MG **Município:** BELO HORIZONTE
**Telefone:** (31)3508-9123 **E-mail:** cephumanos@una.br

**Comentários e Considerações sobre a Notificação:**

O projeto foi finalizado sem intercorrências com os voluntários. Os resultados obtidos foram divulgados em evento científico e as perceptivas de publicação são em uma revista internacional de grande impacto.

**Considerações sobre os Termos de apresentação obrigatória:**

Sem considerações.

**Recomendações:**

Recomendo a aprovação do relatório final.

**Conclusões ou Pendências e Lista de Inadequações:**

Aprovado

**Considerações Finais a critério do CEP:**

**Este parecer foi elaborado baseado nos documentos abaixo relacionados:**

| Tipo Documento | Arquivo | Postagem | Autor | Situação |
|---|---|---|---|---|
| Envio de Relatório Final | relatorio_final.pdf | 25/08/2017 14:50:03 | PEDRO HENRIQUE SCHEIDT FIGUEIREDO | Aceito |

**Situação do Parecer:**

Aprovado

**Necessita Apreciação da CONEP:**

Não

BELO HORIZONTE, 07 de Outubro de 2017

**Assinado por:**
**ANA CRISTINA GOMES SANTOS HOSTT**
**(Coordenador)**

**Endereço:** Rua dos Guajajaras, 175
**Bairro:** Centro **CEP:** 30.180-100
**UF:** MG **Município:** BELO HORIZONTE
**Telefone:** (31)3508-9123 **E-mail:** cephumanos@una.br

## OPINION CONSUBSTANCED OF THE CEP

### DATA OF THE RESEARCH PROJECT

**Title:** COMPARISON OF THE EFFECTS OF INSPIRATORY MUSCLE TRAINING AND AEROBIC TRAINING OF LOWER MEMBERS IN PHYSIOLOGICAL AND FUNCTIONAL PARAMETERS OF PATIENTS IN HEMODIALYSIS

**Researcher:** PEDRO HENRIQUE SCHEIDT FIGUEIREDO

**Thematic area:**

**Version:** 2

**CAAE:** 37412314.5.0000.5098

**Instituition:**

**Main Sponsor:** Universidade Federal dos Vales do Jequitinhonha e Mucuri

### DATA OF THE OPINION

**Number:** 840.370

**Date:** 10/20/2014

**Project presentation:**

Chronic kidney disease (CKD) can be defined as slow and progressive loss of renal function regardless of cause, which in later stages make it impossible for the renal system to develop its regulatory functions. Hemodialysis (HD) is the renal replacement therapy modality most commonly performed in patients with CKD. Although it is a key procedure to control symptoms and increase patient survival, HD is associated with the triggering and maintenance of systemic inflammatory response and neuroendocrine changes, which can affect skeletal muscles, adversely affecting functional capacity and health-related quality of life (HRQL) this population. One of the muscle groups affected by CKD dialysis is the inspiratory musculature. Thus, the training of this muscular group (IMT) was cited as a modality of physical training considered in the rehabilitation programs of these individuals. However, few studies have evaluated the acute and chronic effects of this modality of training in functional, inflammatory and neuroendocrine parameters of this population and its possible influence on the (HRQL). Thus, this study will evaluate the relationship between inflammatory and neuroendocrine factors with functional capacity, muscular strength and HRQL of hemodialysis patients, as well as assessing the acute and chronic effects of IMT

isolated and associated to the aerobic training of the lower limbs on inflammatory, neuroendocrine and functional parameters of this group of patients. To that end, 48 patients will be randomly allocated into four groups: IMT group (IMTG); group aerobic training for lower limbs (ATG); TMI group associated with aerobic training for lower limbs (CTG); and control group (CG). The IMTG volunteers will perform IMT with the Threshold IMT® linear pressure loading device in three sets of 15 repetitions at 50% of maximal inspiratory pressure (MIP) determined by manovacuometry. ATG will perform lower limb training through a portable cycle ergometer, maintaining the pedal cadence at 50rpm and exercise intensity stipulated by the three and seven effort sensation on the modified Borg scale for 30min. CTG subjects will perform IMT prior to lower limb training. All exercise sessions will be intradialitic, performed in the first two hours of the HD session three times a week for 10 weeks. GC volunteers will not suffer any intervention during the study segment. Before and after the execution of the interventions and the follow-up of the CG, the volunteers will be evaluated for: strength of the lower limbs by the Sit-to-Stnd test of 30s; inspiratory muscle strength through the measurement of MIP, through an analog manovacuometer; health-related quality of life by the KDQOL-SF dialysis-specific questionnaire; functional capacity by measuring the distance and direct measurement of the consumption of peak oxygen during the Shuttle Walk Test; blood collection to measure the concentration of inflammatory markers (IL-6, TNF- and PCR); and collection of saliva to measure salivary cortisol concentration. All evaluations will occur on dialysis days, just prior to the HD sessions. To characterize and compare the acute effects of interventions on concentration of markers inflammatory and salivary cortisol concentrations, subjects will have blood and saliva collected before and after the HD session in which the first session of the exercise programs (ATG, GTMI, CTG) or the GC is applied.

**Search Objective**

Primary Objective:

To evaluate the relationship between inflammatory and neuroendocrine factors with functional capacity, muscle strength and HRQoL of hemodialysis patients, as well as to evaluate the acute and chronic effects of IMT alone and associated with lower limb aerobic training in inflammatory, neuroendocrine and functional parameters of this group of patients.

Secondary Objective:

- Evaluate the functional capacity - Evaluate the quality of life related to health - Evaluate the strength respiratory muscle

Analyze the serum concentration of cytokines and C-reactive protein • Analyze the concentration and pattern of daily variation of salivary cortisol • Evaluate the skeletal muscle strength of the lower limbs • Evaluate the associations between these measures • Compare the effects of aerobic training and IMT applied in isolation and associated with the variables described above; - Compare the acute effects of aerobic exercise and IMT applied in isolated and associated serum concentrations of cytokines and C-reactive protein and salivary cortisol.

**Risk and Benefit Assessment:**

Risk:

The risks associated with volunteer participation may be discomfort or embarrassment in responding to the evaluation form and questionnaires, which will be mitigated by being professionally conducted after the researchers' training and in a private setting. The collection of blood causes pain and discomfort

during collection. However, this procedure is part of the clinical follow-up and daily routine of the HD session of these patients. In addition, it will be conducted by a trained professional from the HD industry itself. The risks with the submaximal stress test will be minimized by being applied by trained personnel,

in the presence of a physician and with emergency support. The risks and discomforts induced by the exercise sessions will be minimized, since the intensity of the training will be prescribed in an individualized way, the exercise sessions will be supervised by a trained professional in the presence of medical and emergency support.

Benefits:

The benefits associated with voluntary participation include non-routine exams and assessments: knowledge of data related to cardiovascular risk factors, such as physical inactivity, body mass index, cardiovascular measures and exercise responses, heart rate response to stimuli and other variables collected in the anamnesis, thus establishing a preventive strategy. All volunteers will receive individual records with the results of the evaluation. In addition, the benefits of aerobic training in cardiovascular risk factors, increasing survival, and improving HRQoL in this population are already widely reported in the scientific literature. It is worth mentioning that this type of training is contemplated in two of the three training protocols proposed in this study. However, after reviewing the results, all volunteers will undergo the physical training program that triggers more satisfying responses.

**Comments and Research Considerations:**

The project has justification and relevance. There is no doubt about the relevance and scientific value of the study proposed. Regarding ethical aspects, the project evidences the need for the free consent term and is attached. It records the presence of the required commitments of the researcher and responsible institution, the guarantee of the fundamental rights of the research subject (information, privacy, innocuous refusal, withdrawal, etc.). The questionnaire template is also attached to the which allows the analysis of its content and verification of ethical aspects. It is also observed that the project presents viable budget.

**Considerations Regarding Mandatory Filing Terms**

It presents a cover sheet duly signed by the proposing institution, curriculum vitae investigators, execution schedule, cost table, free and informed consent form, script for data collection and authorization to conduct the research.

**Recommendations:**

See the conclusions item.

**Conclusions or pending and list of inadequacies:**

01) presentation of the letter of agreement of Santa Casa and 02) description of the patient follow-up process. According to the researcher, the procedure will be supervised by a trained professional in the presence of a physician and with emergency support. The collegial vote, therefore,by approval.

**Status of Opinion:**

Approved

**Assessment of CONEP's needs:**

No

**Final considerations at CEP's discretion:**

BELO HORIZONTE, November 12, 2014

Signed by;
**ELAINE LINHARES DE ASSIS GUERRA**
(Coordinator)

**Address:** Rua dos Guajajaras, 175
**District:** Centro **CEP:** 30.180-100
**UF:** MG **Município:** BELO HORIZONTE
**Tel:** (31)3508-9110 **E-mail:** cephumanos@una.br

MINISTÉRIO DA EDUCAÇÃO
UNIVERSIDADE FEDERAL DOS VALES DO JEQUITINHONHA E MUCURI

UFVJM

# FINAL REPORT FOR RESEARCH PROJECTS

| |
|---|
| Nº CAAE: 37412314.5.0000.5098 |
| TITLE:<br><br>COMPARISON OF THE EFFECTS OF INSPIRATORY MUSCLE TRAINING AND AEROBIC TRAINING OF LOWER MEMBERS IN PHYSIOLOGICAL AND FUNCTIONAL PARAMETERS OF PATIENTS IN HEMODIALYSIS |
| Principal investigator: Pedro Henrique Scheidt Figueiredo |
| LOCATION OF THE RESEARCH: Santa Casa de Caridade de Diamantina, Diamantina-MG |
| Current project stage<br>Date of end of research subjects' enrollment: 12/10/2015<br>Total number of subjects enrolled: 43 |
| Results already presented in congress: X Yes ☐ No<br>EVENT : XVIII Simpósio Internaiconal de Fisioterapia Respiratória e Fisioterapia em Terapia Intensiva. |
| Results already published: ☐ Yes X No<br>REFERENCE : |
| Prospects for publications: PlosOne, in the second half of 2017 |
| Occurrence of adverse effects ☐ Yes X No<br>Description of adverse effects: |
| Comments:<br>The project was finalized without adverse effects to the research volunteers. Of the 43 volunteers enrolled, 31 completed all stages of the study. The project was planned to form four experimental groups, three groups of interventions and one control group. Due to the small number of volunteers able to participate in the research, the control group was extinguished and all volunteers went through the control period, which occurred immediately before the intervention period.<br>The three interventions applied had similar beneficial effects. With the results of the study, the physical exercise was introduced by the research group in the hemodialysis sector in which the work was performed and, to date, remains as a clinical intervention of the unit. Research subjects who chose to continue in physical training still remain practicing supervised physical activity. |

| | |
|---|---|
| Date: 08/18/2017 | |
| Researcher Signature | [signature] |

# OPINION CONSUBSTANCED OF THE ETHICS COMMITTEE

**DATA OF THE RESEARCH PROJECT**

**Title:** COMPARISON OF THE EFFECTS OF INSPIRATORY MUSCLE TRAINING AND AEROBIC TRAINING OF LOWER MEMBERS IN PHYSIOLOGICAL AND FUNCTIONAL PARAMETERS OF PATIENTS IN HEMODIALYSIS

**Researcher:** PEDRO HENRIQUE SCHEIDT FIGUEIREDO

**Thematic area:**

**Version:** 2

**CAAE:** 37412314.5.0000.5098

**Instituition:**

**Main Sponsor:** Universidade Federal dos Vales do Jequitinhonha e Mucuri

**DATA OF THE NOTIFICATION**

**Type of Notification:** Submission of Final Report

**Detail:**

**Date of Shipment:** 08/25/2017

**Justification:**

**Notification Status:** Opinion Issued

**DATES OF OPINION**

**Opinion Number:** 2.230.579

**Presentation of Notification:**

Final report of the project entitled "Comparison of the effects of inspiratory muscle training and aerobic training of the lower limbs in physiological and functional parameters of patients in hemodialysis".

**Purpose of the presentation**

Notify the ethics committee of the finalization of the project and the publications related to the projects.

**Risk and Benefit Assessment:**

The project was finalized without adverse effects to research volunteers

**Comments and Notification Considerations**

The project was finalized without intercurrences with the volunteers. The results obtained were divulged in a scientific event and there is the perceptive of publication in an international journal of great impact.

**Considerations Regarding Mandatory Filing Terms**

No considerations

**Recommendations:**

I recommend approval of the final report.

**Conclusions or Pending and List of Inadequacies:**

Approved

**Final Considerations at the discretion of the ethics committee:**

**This opinion was drafted based on the documents listed below:**

| Type of document | File | Posting | Author | Status |
|---|---|---|---|---|
| Submission of Final Report | relatorio_final.pdf | 08/25/2017 14:50:03 | PEDRO HENRIQUE SCHEIDT FIGUEIREDO | Accept |

**Status of opinion:**

Approved

**Assessment of CONEP's needs:**

No

BELO HORIZONTE, November 12, 2014

Signed by;
**ANA CRISTINA GOMES SANTOS HOSTT**
(Coordinator)
